# Characterization of Genetic Diversity and Genomic Prediction of Secondary Metabolites in Pea Genetic Resources

**DOI:** 10.3390/plants15030357

**Published:** 2026-01-23

**Authors:** Stefano Zanotto, Nelson Nazzicari, Gesine Schmidt, Ulrike Böcker, Francesca Vurro, Antonella Pasqualone, Anne Kjersti Uhlen, Paolo Annicchiarico

**Affiliations:** 1 Department of Plant Science, Faculty of Biosciences, Norwegian University of Life Sciences (NMBU), 1433 Ås, Norway; anne.uhlen@nmbu.no; 2 Council for Agricultural Research and Economics (CREA), Research Centre for Animal Production and Aquaculture, 26900 Lodi, Italy; nelson.nazzicari@crea.gov.it (N.N.); paolo.annicchiarico@crea.gov.it (P.A.); 3 Nofima AS, Norwegian Institute of Food, Fisheries and Aquaculture Research, 1433 Ås, Norway; gesine.schmidt@nofima.no (G.S.); ulrike.bocker@nofima.no (U.B.); 4 Department of Soil, Plant and Food Sciences, University of Bari “Aldo Moro”, 70126 Bari, Italy; francesca.vurro97@gmail.com (F.V.); antonella.pasqualone@uniba.it (A.P.)

**Keywords:** antinutritional factors, genetic resources, grain quality, *Pisum sativum*, HPLC-MS, FT-IR

## Abstract

This study aimed to assess the variation, genetic architecture, and genome-enabled prediction of traits with nutritional and health relevance in 156 pea (*Pisum sativum* L.) accessions of diverse geographic origins. The traits included the total phenolic compounds (TPCs), two saponins (Ssβg, Ss1), sucrose, three raffinose-family oligosaccharides (RFOs), and the in vitro antioxidant activity (AA). An analysis of variance revealed significant effects of regional germplasm pools for all traits. Accessions from West Asia showed the highest TPC and AA levels, while those from the East Balkans and the UK displayed the lowest values. High saponin and RFO concentrations characterized accessions from Germany and the UK. Correlation and PCA analyses highlighted strong associations within compound classes and an overall negative relationship between TPCs/AA and saponins/RFOs. The accessions were clustered into seven metabolically distinct groups, partially reflecting their geographic origin. The linkage disequilibrium decayed rapidly (average of 4.7 kb). A GWAS based on 10,249 SNP markers identified 37 significant SNPs, 35 within annotated genes, associated with the metabolites, indicating a polygenic genetic architecture. Genomic prediction models showed a moderately high predictive ability (>0.40) for all traits except the raffinose content. Our findings can support line selection and the identification of genetic resources with a desired level of secondary metabolites.

## 1. Introduction

The field pea (*Pisum sativum* L.) is a globally important cool-season grain legume that is valued for its high yielding ability [1,2], a moderately high seed protein content reportedly in the range of 23–30% [3,4], and various agronomic benefits, such as nitrogen fixation, that enhance the sustainability of cropping systems [5,6]. An increasing interest for pea production in the European union has been driven by the demand for high-protein feedstuff to replace imported plant protein [7], and by the surge in demand for plant-based foods, driven by health, environmental, and ethical concerns [8,9,10].

Domesticated approximately 10,000 years ago in the Near East, the cultivation of peas gradually expanded across the Mediterranean basin, China, and Northern and Eastern Africa [11]. The genetic diversity of modern cultivars is very narrow [11]. Landraces and old cultivars, although largely available in germplasm collections, remain largely underutilized in plant breeding because of some unfavorable agronomic characteristics (e.g., the leafy trait) and the possibly high content of seed antinutritional factors (ANFs) [12,13]. Information on the diversity of the seed quality traits of large pea germplasm collections is focused largely on primary metabolites, especially the protein content, e.g., [14,15], whereas limited information is available for micronutrients and secondary metabolites. Among the various ANFs found in pea seeds, saponins, raffinose-family oligosaccharides (RFOs), and phenolic compounds are some of the most problematic, with negative effects on digestibility in monogastrics. However, some of these molecules have also been reviewed for their positive health-related effects in human diets [16].

Saponins are a diverse group of secondary metabolites that are widely spread in plant species [17]. Pea seeds contain triterpene saponins such as soyasaponins of the B class (of which soyasaponin I is found in peas) and DDMP saponin (soyasaponin βg), which contribute to bitterness and astringency [18,19,20,21]. Saponins in peas and other grain legumes have also been reported for their damaging effect on cell membranes [22]. Their content in different pea genotypes was found to be related to a biotic stress response [23], confirming the role of these compounds in plant defense mechanisms. Despite the antinutritional constraints associated with their consumption, saponins have also been shown to offer health benefits, including hypocholesterolemic, anti-inflammatory, and anti-cancerogenic effects [24,25]. Variety differences in the saponin content have been reported in peas, yet their variation across broad germplasm collections remains understudied. Several steps of DDMP saponin biosynthesis have been studied in different grain legumes, including peas [26]. An effect of the major gene *BAS1* on the pea DDMP saponin content has been identified [27], and a CRISPR/Cas9 protocol for the development of mutant lines with a reduced DDMP saponin content in pea seeds has been reported [28].

The raffinose family of oligosaccharides (RFOs) includes α-D-galactosides of sucrose that are widely accumulated in pea seeds. The digestive tract of humans and monogastric animals lacks α-D galactosidase, an enzyme that hydrolyzes the α-D (1 → 6)-glycosidic bonds between molecules of galactose moieties present in oligosaccharides. Therefore, RFOs are digested with the involvement of the bacterial microflora homing further sections of the intestine. Excessive amounts of carbon dioxide and hydrogen are produced as a result of sugar decomposition and the fermentation of the released monosaccharides, causing flatulence and discomfort in human and monogastrics [29,30]. In extreme cases, the increased content of RFOs in feed ratios for monogastric livestock can provoke diarrhea and reduce energy use. On the other hand, RFOs have beneficial effects thanks to their prebiotic activity and other biological functions, such as anti-allergic, anti-obesity, and anti-diabetic effects and the prevention of non-alcoholic fatty liver disease [16].

Pea seeds are rich in polyphenols, a group of secondary metabolites primarily localized in the seed coat that have potential health benefits for humans. Polyphenols are known for their antioxidant activity and are involved in plant defense mechanisms [31]. Despite the positive health effects associated with polyphenol consumption, these secondary metabolites are also known as ANFs. In particular, condensed tannins hinder the digestibility of peas in monogastric animals [12] and decrease the iron absorption through chelation [32]. The core of the polyphenol biosynthetic pathway is well conserved across plant species [33]. The phenolic profiling of peas and other pulses was recently performed through liquid chromatography and mass spectroscopy (LC-MS) [32,34]. The variation in pea seed coat polyphenols is associated with flower color, with purple color genotypes having specific classes of polyphenols, known as tannins. White flower genotypes lack these compounds and features; therefore, they have a lower total polyphenol content [35]. The genetic regulation of flower color has been widely studied, notably as a character used by Mendel in the study of inheritance in peas, with two major genes being identified in the pea genome as responsible for the white color mutation [36].

The rapid development of next-generation sequencing technologies has revolutionized the study of genetic diversity, with genotyping by sequencing (GBS) emerging as a cost-effective and efficient approach for large-scale SNP discovery and genome-wide association studies (GWASs). Quantitative trait loci (QTL) and candidate genes were identified for pea seed quality traits relative to concentrations of crude proteins, amino acids, minerals, and fibers, as well as protein digestibility [15,37,38,39,40,41,42,43]. Genomic prediction models, which integrate phenotypic and genotypic data to estimate the breeding values of untested genotypes [44], have also been envisaged for the selection of traits, such as the protein content, that exhibit polygenic control [40,41,45], given the higher efficiency of genomic selection relative to marker-assisted selection for such traits [46].

While genome-wide association analyses (GWASs) and genomics have been applied extensively for the study of the agronomic traits in peas, secondary metabolite traits remain underexplored within those frameworks. To address these gaps, this study was designed to (i) characterize the seed saponins, RFOs, total phenolic compounds (TPCs), and antioxidant activity (AA) of 156 pea accessions belonging to a collection previously described by [14]; (ii) conduct a GWAS to unveil the genetic architecture of these traits and identify associated genomic loci and potential candidate genes; and (iii) assess the scope and predictive ability of genomic prediction models for these traits.

## 2. Results

### 2.1. Phenotypic Trait Variation

The 156 pea accessions were grouped into 20 germplasm pools that included 19 regional landrace/old cultivar pools and 1 modern cultivar pool (Supplementary Table S1). The mean and range values of the germplasm pools for the seed content of eight secondary metabolites is shown in Table 1. The analysis of variance (ANOVA) showed significant variation among germplasm pools for the content of all traits (Table 1). The results of Tukey HSD mean comparisons are provided in Supplementary Table S2. On average, the highest and lowest contents of TPCs were observed in the accessions from West Asia and East Balkans, respectively. High TPC values also occurred in the accessions from Greece, Afghanistan, and Ethiopia, whereas low values were a feature of modern cultivars and the accessions from the UK and Nepal. The differences between the modern cultivar pool and the Afghan pool were significant (*p* < 0.05). The highest and lowest values of in vitro antioxidant activity (AA) featured the accessions from West Asia and Germany, respectively. The results for the German pool, however, may have been heavily affected by its very small sample size (just two accessions; Supplementary Table S1) relative to other pools. On average, the soyasaponin βg saponin (hereafter referred to as Ssβg) displayed about a 20-fold higher content than the soyasaponin I saponin (hereafter referred to as Ss1). The accessions from Germany and the UK had particularly high values of the former saponin, while those from France exhibited the lowest values. The materials from the UK had by far the highest content of the Ss1 saponin, while those from Georgia and France had the lowest values. Within RFOs, verbascose showed the highest mean value in the data set (8.52 mg/g), followed by stachyose (6.74 mg/g) and raffinose (2.47 mg/g). The mean value for sucrose was 5.90 mg/g. On average, the UK accessions had the highest verbascose and stachyose contents, while those from Georgia and Ethiopia had the lowest content of these compounds. The accessions from China and Ukraine showed the highest and lowest content, respectively, of sucrose. Finally, the accessions from West Asia and North Africa had the highest and lowest content, respectively, of raffinose. Besides showing low TPC values, the germplasm pool that included the modern cultivars was characterized by fairly low AA, an intermediate content of saponin compounds, and fairly low levels of all RFOs (Table 1).

Several landrace/old cultivar germplasm pools (Turkey, Spain, France, UK, Italy, and Greece) exhibited large within-pool variation across traits, as indicated by the mean value of the genetic coefficient of variation (CVg) values; in contrast, generally low within-pool variation was observed for a few landrace/old cultivar germplasm pools (Germany and Russia), as well as for the modern cultivar pool (Supplementary Table S3). On average, the within-pool genetic variation was particularly high for AA and the two saponins (Supplementary Table S3).

The association among traits was investigated through a correlation analysis and a principal component analysis (PCA). The highest correlations were found between metabolites belonging to the same category. In particular, high correlations (≥0.63; *p* < 0.01) were observed between the two saponin traits, between TPCs and AA, and between the RFO compounds stachyose and verbascose or stachyose and raffinose (Table 2).

**Figure 1:**
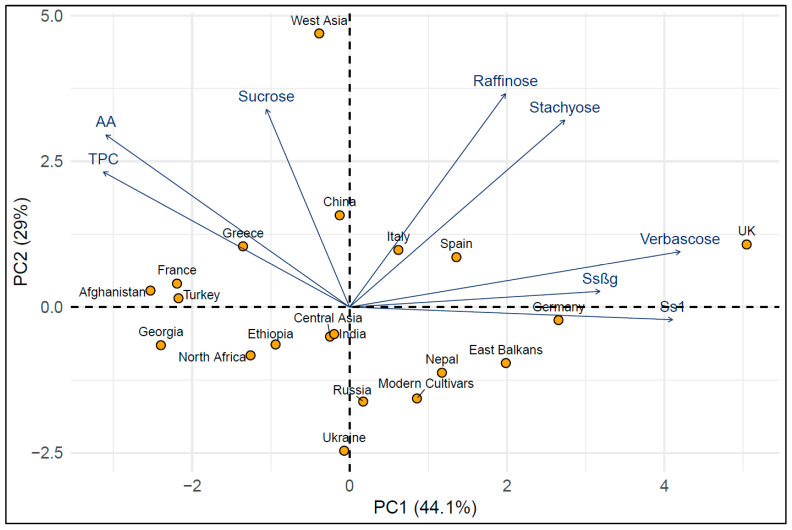
Principal component analysis for traits (standardized values of metabolite content) with nutritional and health relevance observed in 156 pea accessions grouped into 19 landrace/old cultivar germplasm pools and 1 modern cultivar pool. TPCs = total phenolic compounds; AA = antioxidant activity.

The first two axes of the PCA accounted for about 73% of the overall trait variation (Figure 1). The first axis tended to represent a contrast between germplasm pools with high saponin and verbascose contents versus pools with a high phenolic content and high antioxidant activity (Figure 1), suggesting that the latter characters have been selected (or evolved in specific environments under natural selection) differentially compared to saponins and, partially, RFOs. The second PCA axis tended to display a contrast between material with high values of the three soluble sugars (sucrose, raffinose, and stachyose) and, to a lesser extent, a high phenolic content and antioxidant activity, represented especially by the germplasm pool from West Asia, versus material with the opposite characteristics (Figure 1). The PCA plot indicated the similarity of a few landrace/old cultivar germplasm pools with a close geographical origin, such as those from Central Asia and India, or from Russia and Ukraine, or from Italy and Spain. However, the ordination of the germplasm pools in the space of the first two PCA axes was not closely related to their region of origin. The accessions from the UK and, to a lesser extent, those from Germany featured high score values on the first axis, whereas those from Afghanistan, Georgia, Turkey, and France had low score values on this axis (Figure 1). The accessions from West Asia were strongly distinct along the second PCA axis.

**Figure 2:**
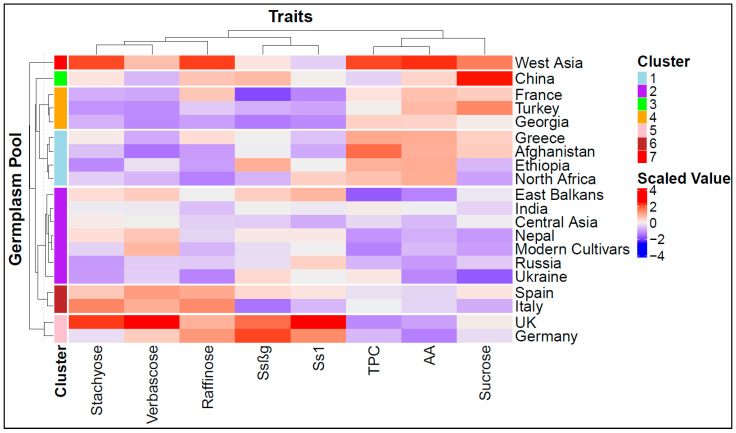
Hierarchical clustering based on the scaled and centered matrix of the content of traits with nutritional and health relevance observed in 156 pea accessions previously grouped into 19 landrace/old cultivar germplasm pools and 1 modern cultivar pool. TPCs = total phenolic compounds; AA = antioxidant activity.

To further explore the similarity of the germplasm pools based on their metabolic profile, we conducted a hierarchical cluster analysis that resulted in the optimal grouping of the pools into seven groups, partly reflecting the geographic similarity of the pools. The heatmap reported in Figure 2 simultaneously visualizes the seven groups and their association with their variation in metabolite contents. One group included the German and UK accessions, featuring high saponin and RFO contents and low TPCs and AA. A second, contrasting group included only the material from West Asia, characterized by high TPCs and AA and a relatively low saponin content. Two other groups (indicated by orange and light blue colors in Figure 2) tended to include germplasm pools of various origins with high TPCs and AA and low RFO and saponin contents. A fifth group included only the germplasm from China, featuring a very high sucrose content. A sixth group included the accessions from Spain and Italy, of which the main characteristic was a higher-than-average RFO content. One last group included the modern germplasm along with landrace/old cultivar groups from various regions of Eastern Europe (Ukraine and East Balkans), Russia, Central Asia, India, and Nepal (Figure 2). Most of this material featured an average or below-average content of all secondary metabolites.

### 2.2. Analysis of Population Structure

**Figure 3:**
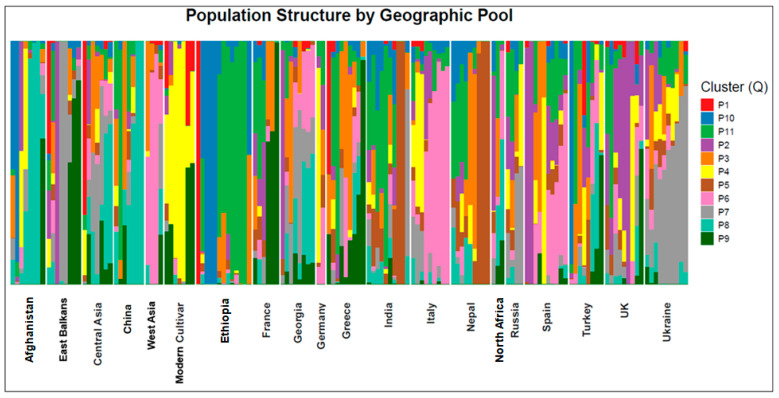
Results of a population structure analysis with Q = 11 performed on 10,249 SNP markers for 151 pea accessions. Each color represents a specific number of genotype groups (Q). Results are displayed for 20 germplasm pools (19 regional pools and 1 comprehending modern cultivar).

Genomic data were available for 151 of the 156 phenotyped accessions. The structure analysis, which was based on 10,249 SNPs, indicated an optimal number of 11 clusters (Supplementary Figure S1; Figure 3). On the whole, the classification of the individual accessions partly reflected the origin and the type (old cultivar/landrace or modern cultivar) of the material. The cluster with the highest number of accessions (light green in Figure 3) comprised most of the accessions from Ethiopia, and several from India. The second most numerous cluster (dark green in Figure 3) included several accessions from France and East Balkans and other single accessions from other pools. Most of the accessions from Italy, several from Spain, and two from West Asia belonged to a third cluster (pink in Figure 3). A fourth cluster (gray in Figure 3) included most of the accessions from Ukraine, two from Russia, and two from East Balkan. Most of the accessions from China and Afghanistan, as well as two from Central Asia, were grouped into an additional cluster (light blue in Figure 3). Three accessions from India and two from Nepal were assigned to a separate cluster (brown in Figure 3). Most of the modern cultivars were grouped into another cluster (yellow in Figure 3), which also included a few European accessions. A total of 62 accessions were classified as admixed. Pairwise *Fst* values among the clusters ranged from 0.03 to 0.14 (mean = 0.07), indicating low to moderate genetic differentiation among the inferred genetic groups.

### 2.3. Linkage Disequilibrium Decay and Genome-Wide Association Study

The linkage disequilibrium (LD) decay was very fast in all chromosomes (Supplementary Figure S2). The distance at which the LD was likely due to physical linkage averaged 4669 bp, with a range from 3390 to 5846 bp.

**Figure 4:**
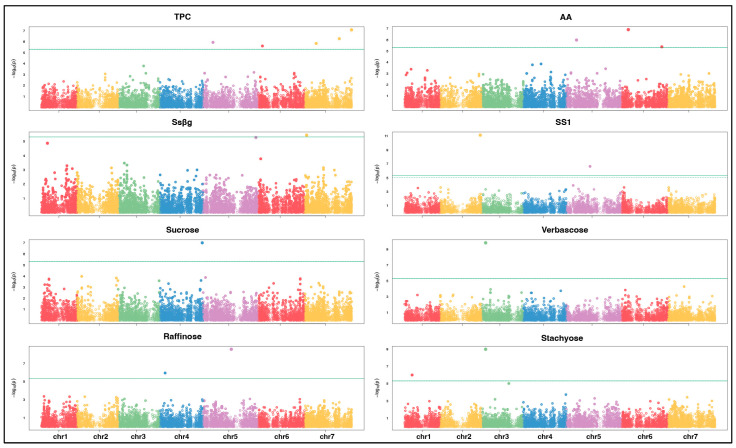
Manhattan plots showing the association scores of 10,249 SNP markers mapped in the seven pea chromosomes, with traits with nutritional and health relevance observed in 151 pea accessions. The green continuous line indicates the Bonferroni threshold of significance at 5%. The figure shows the results of the GWAS conducted with the BLINK model. TPCs = total phenolic compounds; AA = antioxidant activity.

A GWAS was carried out for each trait according to two models, namely BLINK and FarmCPU, selecting the significant SNPs according to the Bonferroni threshold at *p* = 0.05. Figure 4 reports the Manhattan plot results for BLINK, while those for FarmCPU are reported in Supplementary Figure S3. At least one significant SNP was identified for every trait. Three SNPs on chromosome 7, one on chromosome 6, and one on chromosome 5 were identified for TPCs by the BLINK model. One SNP on chromosome 5 was found for antioxidant activity by both BLINK and FarmCPU. For this trait, BLINK identified two additional SNPs on chromosome 6, whereas FarmCPU identified four additional SNPs, of which two were on chromosome 4, one was on chromosome 3, and one was on chromosome 7. For Ssβg, BLINK identified one SNP on chromosome 7 and a second one on chromosome 5 just below the threshold of significance, whereas FarmCPU identified one SNP in each of chromosomes 1, 3, 5, 6, and 7. For Ss1, BLINK and FarmCPU identified the same two SNPs on chromosomes 2 and 5, while FarmCPU identified two additional SNPs on chromosomes 1 and 2. For verbascose, FarmCPU identified six SNPs (one on chromosome 1 and three and two on chromosomes 5 and 7, respectively), while BLINK identified one SNP on chromosome 3. Interestingly, the same SNP was also significant for stachyose according to both BLINK and FARMCPU. For the same trait, FarmCPU identified one additional SNP on chromosome 3 (found just below the threshold of significance from BLINK), one on chromosome 1, and two on chromosome 7. For raffinose, BLINK identified two SNPs on chromosomes 4 and 5. Finally, for sucrose, one SNP was identified by the BLINK model on chromosome 4. Out of a total of 37 different significant SNPs identified by the GWAS, 35 were located within annotated genes, one was within the threshold of LD decay performed in our analysis, and one was not in the vicinity of any annotated gene in the pea genome. The list of the significant SNPs and their putative associated candidate genes are provided in Supplementary Table S4.

### 2.4. Genome-Enabled Prediction

**Figure 5:**
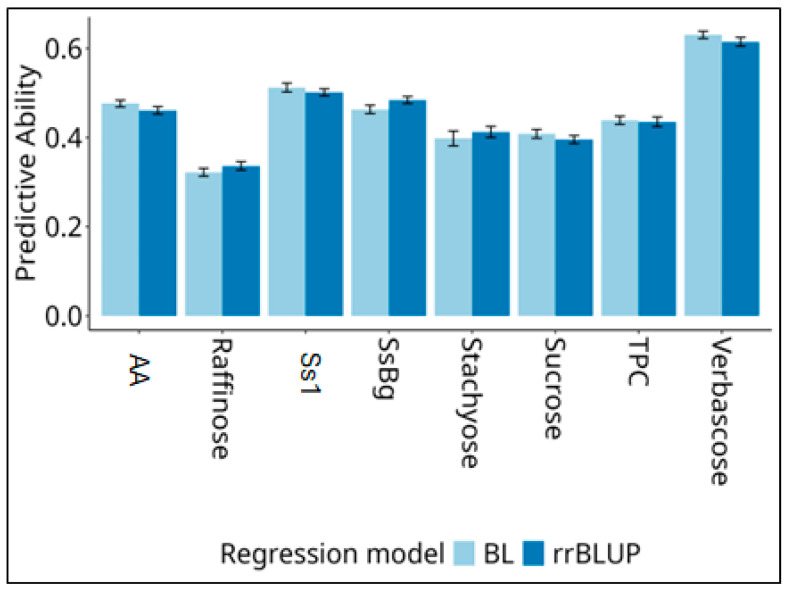
Predictive ability (as correlation between predicted and observed values) and its 95% confidence intervals for genomic prediction of traits with nutritional and health relevance observed in 151 pea accessions according to two statistical models based on 10,249 SNP markers. Predictions based on a 10-fold cross-validation scheme were repeated 50 times. TPCs = total phenolic compounds; AA = antioxidant activity.

The predictive ability of two statistical models (rrBLUP and Bayesian lasso) envisaged for genome-enabled prediction is reported in Figure 5 for each of the eight traits. The Bayesian lasso exhibited a greater predictive performance than rrBLUP for five traits out of eight, but the difference between the models was always modest. Considering the best-predicting model, we observed a high predictive ability (>0.6) for verbascose content and a moderately high predictive ability (>0.4) for all other traits except the raffinose amount.

## 3. Discussion

Our study provides an assessment of the extent of pea genetic variation for eight important quality-related traits that is unprecedented with respect to the number and the diversity of origin of the germplasm under study. Its results indicate a significant and considerably large variation for all traits, which was partially associated with the geographic origin of the accessions.

The range of TPC values was between 0.35 and 1.07 mg GAE/g (Table 1), aligned with the values reported for 100 pea accessions (0.49–1.28 mg GAE/g) [47], but lower than the range reported for ten pea varieties from China (0.66–2.66 mg GAE/g) [48]. The AA values, ranging from 0.16 to 2.40 µmol TE/g, were lower than those reported for 81 pea genotypes [49], which ranged from 0.19 to 3.97 µmol TE/g. Furthermore, the results were significantly inferior to the range of 6.06–12.49 µmol TE/g found in 10 pea varieties [48]. Interestingly, AA was the trait with the highest average CVg across germplasm pools, suggesting wide margins for its improvement through breeding.

The significant differences in the TPC content observed between modern varieties, mostly with white flowers and light-colored seeds, and landraces, particularly those with dark-colored seeds from Afghanistan, were expected according to the known association between these characters [33]. Similar differences in the TPC content between modern and old varieties/landraces have also been observed in other species, due to selection based on seed color [50,51]. Dark pigmentation is partly due to the activity of polyphenol oxidase, which induces the enzymatic browning of phenolics into dark-colored quinones [50], and partly to the presence of pigments, namely anthocyanins [49,52].

Phenolic compounds, which include phenolic acids and flavonoids, stand out as extensively studied functional components of pea seeds [53]. These compounds, particularly the condensed tannins, were traditionally considered ANFs because of their negative effects on protein digestibility in monogastric animals [54], but they have lately been reconsidered as valuable antioxidants able to effectively inhibit free radicals and to prevent oxidative reactions at the cellular level. In addition, phenolic compounds play a pivotal role in modulating the gut microbiota by promoting the growth of beneficial species and inhibiting harmful bacteria [55], and they exhibit a range of biological activities, including anti-diabetic, anticarcinogenic, cardioprotective, and anti-neurodegenerative effects [51,56]. Hence, the consumption of phenolic-rich peas goes beyond a merely nutritional function, offering various potential health benefits [53,55,56]. A highly positive correlation was observed between the TPCs and AA, as reported earlier [57]. The AA of the TPCs is derived from their chemical structure, characterized by a hydroxyl group (-OH) on an aromatic ring, which allows them to neutralize harmful free radicals by donating a hydrogen atom or an electron while being stabilized by resonance, thus interrupting oxidative chain reactions. However, a previous study that included peas and other pulses [32] highlighted a different contribution to the AA of different types of polyphenols, suggesting to target specific compounds rather than the general TPCs by breeding work. Indeed, the increase in these compounds may result in negative effects, such as iron chelation and protein precipitation. The complexity of the phenolic compounds’ biosynthetic pathway in grain legumes was recently highlighted by a study on faba beans [58]. This study indicated how different genotypes with the same genetic background at the low tannin gene locus (*zt*) had very different phenolic profiles, and how this was partially affected by the environment, suggesting that several genes regulate the secondary branches of this pathway. A qualitative characterization of the phenolic compounds of the accessions would represent a useful follow-up of this study.

The current overall variation for RFOs agrees substantially with previous studies that analyzed the content of sucrose and RFOs in Spanish breeding lines [59] and a pea collection from the Polish gene bank [29]. Stachyose and verbascose consistently accounted for the highest total amount of RFOs, although the maximum absolute values that we found (up to 16.20 for verbascose and 10.30 mg/g for stachyose) were lower than those reported in those studies. We identified low variation in the raffinose content across germplasm pools as well as among individual accessions. This result may partly depend on the generally low raffinose content, since FT-IR-based models are less effective at detecting trait variation for metabolites with a low concentration. The authors observed a high correlation between stachyose and verbascose that is consistent with earlier findings and the fact that these compounds are synthesized through the same biosynthetic pathway, although different enzymes are active in the biosynthesis of verbascose in different pea genotypes [30]. No earlier studies have reported a negative correlation of verbascose content with AA or the TPC content in legumes, and further investigations would be needed to further verify this relationship and explore its biochemical basis. In contrast, raffinose was reported to be a moderate ROS scavenger in vitro [60], while we observed a non-significant correlation of this compound with AA.

Using the information on seed morphology and seed color previously reported for our panel [41], we verified through *t*-tests that the variation in the RFO content was significantly associated with major seed traits. The accessions with a transparent seed coat (recessive allele) showed higher RFO levels than those with a colored testa (17.5 vs. 16.1 mg/g); those with a wrinkled seed (recessive allele) had a higher RFO content compared with smooth-seeded lines (23.5 vs. 16.4 mg/g); and those with a green cotyledon (recessive allele) accumulated more RFOs than those with yellow or dark cotyledons (20.2 vs. 16.5 mg/g). These findings are consistent with previous reports indicating that seed morphology and seed color are linked to variation in the oligosaccharide composition in peas, with substantially lower RFO levels observed in lines carrying the dominant alleles controlling seed shape, seed coat color, and cotyledon color [29]. Similarly, an association of the seed coat with the concentration of soluble sugars was reported [59].

The observed variation in the saponin content was wide, both for Ssβg (often reported as DDMP saponin in peas; range of 0.04–1.07 mg/g), which represented the main compound, and Ss1. However, a wider range of the total saponin concentration, namely 0.8–2.5 mg/g, was previously reported [19]. Advances in gene editing such as CRISPR/Cas9 might help to quickly introduce the low-saponin mutation in elite pea germplasms [28], but regulation issues might be a limiting factor for practical applications of such technologies. Therefore, the identification of accessions, such as those from the French and Georgian pools, with a considerably lower saponin content than that of modern cultivars is remarkable from a breeding perspective, encouraging the selection for reduced saponins through the crossing of these genetic resources with elite lines.

The analysis of the population structure based on SNP data indicated a partial association between genetic diversity and the geographical origin of the material. Our results agree with those in [41] based largely on the same plant material. Modern cultivars tended to be genetically distinct from landrace germplasm pools in the structure analysis. Interestingly, the metabolic profile of the set of modern cultivars was characterized by an intermediate content of saponins and RFOs and a fairly low content of TPCs and AA. Given the effects on taste and palatability of phenolics and saponins (often resulting in bitterness) and the negative effects on the digestibility of RFOs, selection for quality has likely resulted in a generally low content of these metabolites in modern cultivars. However, considering the positive health effects of these compounds indicates scope for improvement in the nutritional profile of modern germplasms by the introgression of useful variation from old varieties and landraces through a more targeted breeding approach to quality.

The GWAS identified various SNPs associated with each of the focus traits, a result that supported their polygenic control. The investigation of gene ontology (https://plants.ensembl.org/) suggested that most of the candidate genes containing or in LD with the significant SNPs seem to play a role in cell metabolism, the general stress response, or DNA transcription regulation. Among the genes that were characterized by more specific functions, *Psat5g137080*, associated with Ss1 saponin and identified by both the BLINK and FarmCPU models, encodes an acetyl-CoA carboxylase protein that plays a role in the fatty acid biosynthetic process [61]. The Ss1 saponin is a lipid-derived molecule to which a sugar moiety is added in a later stage of the biosynthetic pathway to give it both hydrophobic and hydrophilic properties. *Psat7g192120*, here associated with the TPCs, has an orthologue in *Arabidopsis thaliana* (*NCRK*) that is known to code for a cysteine-rich receptor-like kinase (*RLK*) that is involved in plant signaling pathways, including those responding to stress and pathogen attacks [62]. Phenolic compounds are well known to be active in the plant stress response, and their accumulation in plant organs is often enhanced in response to both biotic and abiotic stresses. The *Psat1g013760* gene was associated with verbascose and codes for a histidyl-tRNA synthetase protein. The *Arabidopsis thaliana* orthologue of this gene (*HRS1*) encodes a transcription factor involved in integrating nitrate and phosphate signaling to regulate root development and seed germination [63]. RFOs were shown to contribute to the control of seed germination in *Arabidopsis* [64] and were correlated with an enhanced seed vigor in *Zea mays* [65]. Our inability to identify genes with a clear disruptive function in the biosynthetic pathways of the secondary metabolites, and the identification of several genes with broad metabolic roles, indirectly supports the conclusion that these traits are under polygenic control. Such a result would support the usefulness of genome-enabled prediction models for breeding line selection and/or the identification of useful genetic resources.

Although the Bayesian lasso is theoretically more suitable than rrBLUP for the genomic prediction of traits that are not controlled by many genes [66], its advantage occurred only for five of the eight secondary metabolites and, anyway, the two statistical models had a similar predictive ability for all traits. A similar predictive ability of the two models was reported earlier for other pea traits that, although polygenic, are expected to be less genetically complex than the crop yield, such as the protein content, onset of flowering, seed weight, frost resistance, and tolerance to rust [41,67,68,69]. The moderately high predictive ability (>0.4) observed for all traits except the raffinose content has a high perspective interest for the selection of breeding lines or the identification of genetic resources with a desired level of secondary metabolites. The lower predictive ability for raffinose might result from the generally low phenotypic variation identified for this trait by FT-IR. This trait could be considered the least important of the eight secondary metabolites, as it was the RFO with the lowest content in the seed. Our results, combined with earlier results for the same germplasm collection showing a moderate predictive ability (0.55) also for the protein content, would allow a multi-trait genome-enabled selection to be envisaged for a combination of primary and secondary traits. The predictive ability of the current models for reference populations other than the current global germplasm collection (and similar material) is pending verification. The prediction model for the protein content constructed from data on the current germplasm collection displayed a moderate fall of predictive ability (from 0.55 to 0.28) when applied for the prediction of a completely different, genetically narrow reference population of breeding lines evaluated under climate conditions quite different from those used for the germplasm collection [41].

A limitation of this study is represented by the performance of phenotyping under greenhouse conditions and in just one growing environment. Future work should aim to characterize these secondary metabolites in replicated multi-environment field trials, to assess the extent of genotype-by-environment interactions, and to improve the robustness of genetic analyses and prediction models.

In conclusion, the information provided by this study on the extent and geographical pattern of genetic variation, the genetic control, and the genome-enabled prediction of the eight focus traits can contribute to more targeted and efficient breeding efforts aimed to select breeding lines and identify genetic resources with a desired level of secondary metabolites, filling a research gap and meeting the growing demand for pea production with suitable traits.

## 4. Materials and Methods

### 4.1. Plant Material

The study included 156 ecotypes or old cultivars of *P. sativum* subsp. *sativum* subdivided into 19 regional pools, each represented by 4–14 entries, and 7 modern cultivars bred in France (Attika, Dove, Isard, Spirale), Spain (Cigarron, Viriato), or Germany (Santana) (Supplementary Table S1). This collection, which included accessions of field peas intended for dry harvest, was previously described and characterized for agronomic traits [14]. The accessions were kindly provided by curators of pea germplasm collection established at the International Center for Agriculture Research in the Dry Areas (Aleppo), the John Innes Centre (Norwich), INRA-UMR Agroécologie (Dijon), the Agricultural Technological Institute of Castilla and Leon (Valladolid), the Crop Research Institute (Praha), the Institute of Plant Genetics and Crop Plant Research (Gatersleben), and the CNR-Institute of Biosciences and Bioresources (Bari). Curators were asked to select accessions with the aim of adequately representing the germplasm variation that features each regional pool on the basis of the available collecting or morphophysiological data.

Plants were grown in 7.5 L pots filled with peat soil in greenhouses at the Norwegian University of Life Sciences (NMBU) in 2023. Each accession was represented by two pots containing four seeds each. This small number of biological replicates was due to limited seed availability. The pots were randomized in the greenhouse, setting the growing conditions to a 16 h photoperiod, a 300 μmol/ m^−2^ s^−1^ photosynthetic photon flux density (PPFD), and a 21–16 °C day/night temperature. The plants were fertilized once during the experiment with 3 g/pot of NPK (19-4-12) fertilizer and watered regularly to maintain optimal growth. They were progressively harvested when they reached optimal maturation, pooling the seeds belonging to the same accession. Seeds were oven-dried at 60 °C for 24 h before sample preparation for the chemical analyses.

### 4.2. Chemical Analyses

A dried seed sample of 100 g from each accession was milled using a Retsch Twister (Retsch GmbH, Haan, Germany). Flour from the same sample was used for all chemical analyses.

#### 4.2.1. Total Phenolic Compounds and Antioxidant Activity

The extraction of the TPCs was carried out according to [70]. In detail, 1 g of the sample was extracted with 5 mL of an 80:20 methanol/water solution (*v*/*v*). The suspensions were submitted to ultrasound (CEIA international S.A., 115/230 Vac 1—50/60 Hz–400 Watt, Viciomaggio, Italy) for 15 min at room temperature, then shaken for 30 min and centrifuged (Thermo Fisher Scientific, Osterode am Harz, Germany) for 10 min at 12,000× *g* and 4 °C. The supernatants were filtered through 0.45 μm nylon filters. The TPCs were then quantified as described in [71] with some modifications. Specifically, 200 µL of the filtered extract was added to 800 µL of deionized water and 100 µL of Folin–Ciocalteu reagent and kept in the dark for 3 min; then, 800 µL of 7.5% Na_2_CO_3_ was added, followed by incubation for 60 min, again in the dark. Spectrophotometric quantification was carried out at 720 nm using a Cary 60 UV–Vis spectrophotometer (Agilent Technologies, Santa Clara, CA, USA) and the TPCs were expressed as the mg gallic acid equivalents (GAE)/g of the sample. The analysis was carried out in triplicate.

To assess the in vitro AA, the phenolic extracts were submitted to a radical scavenging assay using the 1,1-diphenyl-2-picrylhydrazyl (DPPH) radical, according to [71]. A 0.08 mM solution of DPPH in ethanol was freshly prepared. For the analysis, 50 µL of the extract was added to 950 µL of DPPH solution. After 30 min of incubation in the dark, the absorbance was read at 517 nm. The results were expressed in µmol of 6-hydroxy-2,5,7,8-tetramethylchroman-2-carboxylic acid (Trolox) equivalents (TE)/g of the sample. The analysis was carried out in triplicate.

#### 4.2.2. Saponins

Two replicates, each consisting of 50 ± 0.2 mg of dried flour in a 2 mL vial, were prepared for each accession. In addition, an extraction blank, i.e., a vial without pea flour, was prepared for each extraction day. The blanks and sample replicates were randomized, and then each was extracted in 500 µL of 80/20 methanol/water (*v*/*v*, 80% MeOH containing 1 µg/mL ginsenoside Rb1 as ISTD). The extracts were shaken for 30 min (1400 rpm, 20 °C), then centrifuged for 5 min (12,000 rpm, 20 °C, Heraeus Fresco21, Thermo Fisher Scientific). After removing 400 µL of the supernatant to a second vial, the pellet was resuspended and re-extracted as before in 500 µL of 80% MeOH (without ISTD). Then, 500 µL of the supernatant was combined with the previous 400 µL, vortexed, filtered through 0.2 µm regenerated cellulose (RC) syringe filters (Phenomenex Inc., Torrance, CA, USA), and finally diluted to 1:10 in 80% MeOH. A QC sample was prepared by pooling aliquots from each extract. The extracts were analyzed in a newly randomized order and after the injection of the extraction blanks on a Vanquish Horizon UHPLC coupled to an Orbitrap IQ-X iontrap orbitrap mass spectrometer (both Thermo Fisher Scientific). The chromatographic system consisted of an autosampler, a pump, an Ascentis Express C18 column (150 mm × 2.1 mm, 2.7 µm, Merck, Darmstadt, Germany) with a 5 mm guard cartridge maintained at a stable 20 °C in a column compartment, and a photodiode array detector (PDA) recording at 205 nm and 227 nm. The mobile phase was (A) water (0.2% FA) and (B) ACN:MeOH, 3:1 (*v*/*v*, 0.2% FA), with the following gradient: 0 min, 15% B; 10–14 min, 90% B; 14.1–16 min, 100% B; 16.1–24 min, 15% B. The flow was 0.25 mL/min and 1 µL of the sample was injected. The heated ESI spray chamber settings were as follows: spray, −3500 V; sheath gas, 40 au; aux gas, 15 au; sweep gas, 2 au; ion transfer tube, 350 °C; vaporizer, 325 °C. The MS was recorded in full scan mode in the time range of 1.5–16 min and the mass range of *m*/*z* = 500–1700 Da. Saponins were first identified by their molecular ion as well as their tandem mass spectra (MS2) acquired representatively on the QC sample. Isolation and targeted fragmentation (tMS2) were performed on 5 candidate molecular ions (Supplementary Table S5) identified from PDA and MS chromatograms. Fragmentation used collision-induced dissociation (CID) with a 10 ms activation time at normalized collision energies of 30%, 45%, and 60% and a 5 s cycle time. The isolation window was *m*/*z* = 3 Da and the isolation offset was *m*/*z* = +1 Da. The resolution was set to R = 60,000 FWHM (at *m*/*z* = 200) for the full scan and R = 30 000 FWHM for MS2 scans. Three scans were averaged to improve the MS2 spectrum quality. The MS2 spectra were subsequently compared to published data. Individual saponins were then quantified based on their parental extracted ion chromatogram, corrected for ISTD, as soyasaponin-I equivalents based on an 11-point standard dilution series (0.005–5 µg/mL). The QC sample was analyzed after every 10th sample injection. Quantification was performed in TraceFinder v.5.1 (Thermo Fisher Scientific) and corrected for sequential extraction loss.

#### 4.2.3. HPLC Quantification of Sucrose and RFO for FT-IR Calibration

For each genotype, one sample consisting of 50 ± 2 mg of finely ground pea flour was weighed into 15 mL polypropylene centrifuge tubes. The samples were extracted in 10 mL of 50% (*v*/*v*) ethanol containing 50 µg/mL melibiose as an internal standard (prepared from a 1 mg/mL stock solution in Milli-Q water with 0.02% sodium azide). The tubes were vortexed and subsequently shaken horizontally at 250 rpm and 50 °C for 1 h in an incubator. After extraction, the samples were allowed to stand vertically at room temperature for 15 min and then centrifuged at 4000× *g* for 10 min. The resulting supernatant was diluted to 1:10 (*v*/*v*) with Milli-Q water, filtered through 0.22 µm PVDF syringe filters (Merck KGaA) into HPLC vials, and used for a chromatographic analysis.

Soluble sugars were quantified by high-performance anion-exchange chromatography coupled with pulsed amperometric detection (HPAEC–PAD) using a Dionex ICS 5000+ system (Thermo Fisher Scientific, USA) equipped with an AS-AP autosampler, ICS 5000+ SP pump, and ICS 5000+ DC column oven and detector compartment. The detection system consisted of a pulsed amperometric detector with a gold working electrode and an Ag/AgCl reference electrode, operated using the following waveform: 400 ms at 0.11 V; ramp 10 ms from 0.10 V to −2.00 V; 10 ms at −2.00 V; ramp 10 ms from −2.00 V to 0.60 V; ramp 10 ms from 0.60 V to −0.10 V; and 60 ms at −0.10 V. Separation was achieved on a CarboPac PA-1 analytical column (Thermo Fisher Scientific) with a CarboPac PA-1 guard column, maintained at 25 °C. The mobile phase consisted of (A) MilliQ-water, (B) 200 mM NaOH, and (C) 100 mM NaOH, 500 mM Na-acetate using the following A/B/C (*v*/*v*) gradient program: 0–5 min, 49:49:2; 28–31 min, 48:48:4; 31–34 min, 0:0:100; followed by re-equilibration at start conditions. The injection volume was 25 µL, and the flow rate was 1 mL min^−1^. Quantification was performed using external calibration with mixed sugar standards (sucrose, raffinose, stachyose, and verbascose) prepared in the same solvent system, with melibiose as the internal standard.

#### 4.2.4. FT-IR-Based Modeling for Sucrose and RFOs

The content of soluble sugars, namely sucrose, raffinose, verbascose, and stachyose, was estimated by Fourier transform infrared spectroscopy (FT-IR) with partial least squares (PLS) calibration models, using the HPLC results for individual sugars as a reference.

For the FT-IR measurements, a Bruker Invenio spectrometer (Bruker Optics, Billerica, MA, USA) equipped with a Pike MIRacle diamond crystal ATR accessory with a deuterated triglycine sulfate (DTGS) detector was used for data collection. The spectra were recorded in the region between 4000 and 600 cm^−1^ with a spectral resolution of 4 cm^−1^ based on 32 scans. Before each sample, a background spectrum of the empty ATR crystal was recorded to compensate for water vapor and CO_2_. Three replicates were measured for each sample. All the FT-IR spectra were imported to the Aspen Unscrambler, version 14 (AspenTech Inc., Bedford, MA, USA), and the spectra were processed by applying a Savitzky-Golay 2. derivative with a 2.degree polynomial and a window size of 9. PLS models were calculated from the average spectra for the spectral range of 1800–700 cm^−1^, using the individual sugars as a reference. This resulted in the following models: for sucrose, the root mean square error of cross-validation was RMSECV = 0.9 and R^2^ = 0.83 (7-factor model); for verbascose, RMSECV = 1.27 and R^2^ = 0.77 (6-factor model); for raffinose, RMSECV = 0.26 and R^2^ = 0.77 (7-factor model); and for stachyose, RMSECV = 1.2 and R^2^ = 0.46 (3-factor model). For further statistical analyses, the sugar content from three individual replicate spectra was predicted from these models.

### 4.3. Statistical Analyses

For each trait, a linear model with the random effects accession and replicate was fitted using the lmer() function from the lme4 package [72] of the R software 4.3.3 (2024-02-29) with the following formula:

$$Y_{ij}=µ+u_{i\;}+v_{j}+\;\epsilon_{ij\;}$$

where $Y_{ij}$ is the phenotype; µ is the overall mean; $u_{i\;}$ represents the random effect of the *i*-th genotype; $u_{i\;}∼N(0,\sigma_{u}^{2})$; $v_{j}\;$ represents the random effect of the *j*-th replicate; $v_{j}∼N(0,\sigma_{v}^{2})$; and $\epsilon_{ij\;}∼N(0,\sigma_{\epsilon }^{2})$ is the residual error term. The best linear unbiased predictor (BLUP)-adjusted means for each accession were calculated by adding the random effect deviations to the overall intercept and used as the input data for the subsequent correlation analyses, multivariate analyses, and genome-wide association studies (GWASs). For the TPCs, AA, and soluble sugars, the models were fitted using data from three analytical replicates per accession, while two replicates per accession were used for saponins.

The response variables were evaluated for normality using both visual inspection (histogram and Q–Q plot of model residuals) and the Shapiro–Wilk test. Although, for some traits, the Shapiro–Wilk test indicated departures from perfect normality (*p* < 0.05), the visual assessment suggested approximately normal distributions without major deviations, supporting the suitability of the data for a mixed model analysis.

An analysis of variance (ANOVA) that included the fixed factor of the germplasm pool and the random factors of the accession within the germplasm pool and the replicate assessed the variation between the 20 germplasm pools (hence, holding the accession within the germplasm pool as the error term for a germplasm pool comparison).

To assess the magnitude of genetic variation within each germplasm pool, the variance between accessions of each germplasm pool was estimated and the genotypic coefficient of variation (*CVg*) was calculated as the square root of the genotypic variance divided by the trait mean value for the relevant pool.

Pearson’s correlation coefficients were estimated from BLUPs of individual accessions to test the association among traits. Multivariate patterns of variation among germplasm pool accessions were investigated by a principal component analysis (PCA) and a hierarchical clustering analysis (HCA). These analyses were performed on the BLUP matrix of the germplasm pool by trait after trait standardization to a zero mean and unit variance. The PCA and HCA were performed with the PCA() and hclust() functions of factormineR [73] and the stats package function of R, respectively. For the HCA, the distance matrix was calculated using the Euclidean distance with the dist() function of the stats package according to the Ward clustering algorithm. The optimal number of clusters was chosen both through a visual assessment of the dendrograms and the silhouette method after generating the relative plot with the package cluster() [74]. Finally, a heatmap was created with the Heatmap() function from the ComlexHeatmap package [75], to simultaneously visualize the HCA results for the metabolites and accessions. All other statistical analyses were performed with the R software 4.3.3 (2024-02-29).

### 4.4. GBS, SNP Calling, Marker Filtering, and Imputation

Information on DNA isolation and GBS-based genotyping can be found in [76]. SNP calling was performed using the Legpipe2 pipeline [77] with the default settings for diploid species, with a preliminary filtering for mapping quality (MQ < 40). For alignment, we used the *Pisum sativum* L. (2n = 14) reference genome, version 1a [78]. The whole set was filtered for a minor allele frequency (MAF) > 5% and a missing rate < 50% and imputed with the k-nearest neighbors (KNN) method. This process retained 10,249 SNP markers.

### 4.5. Linkage Disequilibrium, Population Genetic Structure, and Genome-Wide Association Study (GWAS)

Genomic data were available for only 151 of the 156 accessions characterized with phenotypic analyses; therefore, only 151 accessions were considered for the following genomic analyses. The linkage disequilibrium (LD) for each single chromosome was calculated using only the SNP markers with a known chromosomal position relative to the reference genome [78]. The LD was estimated as the squared allele frequency correlations (r^2^) for each pairwise combination of markers distanced within 100 Kbp with the pcor.shrink () function of the package corpcor in R studio. The LD was then plotted against the genomic distance between marker pairs and the LD decay was visualized with the LOESS regression model. The LD decay was estimated as the point where the fitted curve reached half of its maximum value [79].

The population structure was analyzed with the snmf() and Q() functions of the R package LEA [80], which output the results in a similar way as the STRUCTURE software 2.3.4. (2012-07-01), although it employs different algorithms and is indicated as being more accurate for self-pollinating species [41]. The filtered and imputed SNP data, as described in the section above, were used as input in the analyses. The optimal number of genetic clusters was selected after a visual assessment of the cross-entropy parameter plot estimated through cross-validation. Genetic differentiation among the inferred clusters was quantified using pairwise Weir and Cockerham’s *Fst*, calculated with the R package hierfstat [81] based on cluster assignments inferred from sNMF ancestry coefficients.

A GWAS was conducted for the eight phenotypic traits for 151 pea accessions using two models, namely FarmCPU and Blink, using the R package “GAPIT” [82]. The population structure was accounted for by including the first 2 principal components of a PCA conducted on the SNP data (Supplementary Figure S2). The appropriate compensation of the population structure was assessed through a visual examination of quantile–quantile (QQ) plots. For traits that showed an over-compensation of the population structure (Ssβg and Ss1), GWAS models with a reduced number of principal components (1 or 0) were performed. Significant SNPs were selected according to the Bonferroni threshold at 5%, which was preferred to the less conservative false discovery rate (FDR), to minimize false-positive associations in a relatively small sample size. Candidate genes associated with the significant SNPs and their putative function were searched with the pea genome browser (https://www.pulsedb.org/), investigating chromosomal regions corresponding to the chromosome-specific LD extent flanking the significant SNP.

### 4.6. Genomic Prediction

The SNP markers retained after SNP calling and filtering were employed to build GS models for the eight phenotypic traits. We compared two regression models, namely ridge-regression BLUP (rrBLUP) and the Bayesian lasso. The former model [83] assumes a linear mixed additive model in which each marker is assigned an effect as a solution of the following equation:

y=1µ+Wq+E

where *y* is the vector of the observed phenotypes, µ is the mean of *y*, *W* is the genotype matrix (e.g., {0,1,2} for biallelic SNPs), *q* ∼ N (0, ${I\sigma }_{q}^{2}$) is the vector of marker effects, and *ε* ∼ N (0, ${I\sigma }_{e}^{2}$) is the vector of residuals. This model, which is solved in a restricted maximum likelihood (REML) context, assumes that the effects of all loci have a common variance, making it suitable for traits influenced by a large number of minor genes. According to a Bayesian context, the Bayesian lasso assigns different prior densities to marker effects, with a strong shrinkage for regression coefficients of marker effects with small values [84]. Predictive ability values were computed as Pearson’s correlation between the observed phenotypic values and the breeding values predicted by the regression model. We implemented a 10-fold cross-validation scheme repeated 50 times for numerical stability. The reported values are the resulting averages. All regressions and cross-validations were implemented using the GROAN R package [85].

## Figures and Tables

**Table 1:** Mean and range values, and analysis of variance (ANOVA) F-value for mean comparison, for seed content of secondary metabolites with nutritional and health relevance observed in 156 pea accessions grouped into 19 landrace/old cultivar germplasm pools and 1 modern cultivar pool. GAE = gallic acid equivalents; TE = 6-hydroxy-2,5,7,8-tetramethylchroman-2-carboxylic acid (Trolox) equivalents.

Germplasm Pool	Total Phenolic Compounds (mg GAE/g)	Antioxidant Activity (µmol TE/g)	Ssβg Saponin (µg/g)	Ss1 Saponin (µg/g)	Sucrose (mg/g)	Raffinose (mg/g)	Stachyose (mg/g)	Verbascose (mg/g)
Afghanistan	0.80 (0.55–1.00)	1.35 (0.43–2.34)	500 (240–712)	21.4 (10.9–34.1)	6.36 (4.44–8.79)	2.16 (1.49–2.77)	6.44 (5.34–7.87)	6.73 (3.72–9.86)
Central Asia	0.65 (0.52–0.72)	0.83 (0.53–1.29)	466 (263–754)	21.6 (16.2–29.4)	5.87 (4.32–10.0)	2.35 (1.77–3.47)	6.79 (5.93–8.85)	8.54 (6.24–10.4)
China	0.65 (0.57–0.74)	1.17 (0.85–1.37)	571 (382–718)	27.1 (14.3–41.4)	8.08 (4.71–10.5)	2.66 (2.00–3.34)	6.84 (5.99–8.91)	7.70 (4.59–9.78)
East Balkans	0.54 (0.35–0.72)	0.62 (0.16–1.13)	546(307–749)	32.3 (13.2–53.8)	5.80 (4.09–8.85)	2.47 (1.52–3.61)	6.89 (4.89–8.97)	9.13 (6.75–12.6)
Ethiopia	0.74 (0.48–0.83)	1.37 (0.85–2.14)	584(273–961)	26.9 (11.2–36.9)	5.32 (2.38–7.99)	2.17 (1.60–2.64)	6.08 (5.02–7.09)	8.29 (6.10–10.2)
France	0.69 (0.49–0.84)	1.27 (0.70–1.60)	331 (212–553)	18.7 (10.4–31.1)	6.35 (3.75–11.7)	2.66 (1.88–4.22)	6.31 (5.41–7.88)	7.45 (5.46–10.6)
Georgia	0.70 (0.56–0.85)	1.18 (0.55–1.93)	381(154–624)	19.1 (9.40–30.3)	5.92 (2.57–8.38)	2.17 (1.47–2.82)	6.33 (4.94–7.67)	7.04 (3.55–9.99)
Germany	0.62 (0.60–0.65)	0.60 (0.49–0.71)	696 (690–702)	36.1 (34.3–38.0)	5.70 (4.91–6.49)	2.85 (2.72–2.99)	6.62 (6.49–6.76)	9.15 (8.56–9.73)
Greece	0.75 (0.53–1.07)	1.37 (0.86–2.10)	501 (155–801)	23.4 (6.6–36.1)	6.29 (3.79–10.7)	2.55 (1.77–3.85)	6.79 (5.94–8.32)	7.48 (2.79–10.7)
India	0.68 (0.46–0.83)	1.03 (0.34–2.14)	503 (290–677)	26.3 (12.4–49.0)	5.59 (4.09–8.76)	2.29 (1.86–3.01)	6.72 (5.75–7.43)	8.42 (6.34–10.7)
Italy	0.67 (0.57–0.80)	0.93 (0.69–1.16)	373 (124–741)	22.6 (9.61–34.5)	5.19 (2.94–9.10)	2.91 (1.87–4.12)	7.56 (5.87–10.53)	9.70 (6.18–12.8)
Nepal	0.59 (0.43–0.90)	0.79 (0.38–1.83)	512 (340–919)	27.7 (19.6–43.6)	4.98 (4.09–7.66)	2.37 (1.80–3.23)	6.88 (5.24–8.06)	9.26 (8.02–10.1)
North Africa	0.72 (0.59–0.81)	1.36 (0.88–1.74)	440 (404–476)	29.9 (20.5–42.2)	5.06 (4.22–5.78)	2.06 (1.76–2.22)	6.52 (5.59–7.30)	7.65 (7.52–7.73)
Russia	0.62 (0.50–0.70)	0.71 (0.61–0.87)	484 (370–564)	29.8 (23.3–37.2)	5.51 (4.14–6.13)	2.33 (2.02–2.55)	6.18 (5.32–6.92)	8.00 (6.74–10.6)
Spain	0.66 (0.48–0.76)	0.94 (0.42–1.98)	531 (43–768)	28.1 (2.10–44.7)	6.04 (2.48–10.1)	2.79 (1.85–3.62)	7.07 (5.34–9.56)	10.00 (8.57–14.2)
Turkey	0.67 (0.52–0.83)	1.30 (0.71–2.03)	436 (101–819)	21.0 (6.17–41.9)	7.20 (4.93–11.3)	2.33 (1.84–3.41)	6.14 (4.77–7.41)	7.06 (1.47–10.8)
UK	0.58 (0.49–0.71)	0.73 (0.16–1.56)	658 (284–1007)	47.3 (27.9–83.2)	5.97 (3.79–9.45)	2.75 (2.01–3.81)	7.99 (5.10–10.3)	11.74 (6.28–16.5)
Ukraine	0.68 (0.57–0.98)	0.63 (0.34–1.42)	532 (358–754)	26.9 (14.0–40.5)	4.33 (2.98–6.13)	2.07 (1.51–2.56)	6.19 (5.57–7.51)	8.02 (6.23–10.1)
West Asia	0.83 (0.67–0.92)	1.82 (1.56–1.97)	517 (333–694)	24.5 (18.6–34.7)	7.32 (5.80–8.84)	3.16 (3.00–3.24)	7.92 (7.30–8.75)	9.40 (8.24–10.5)
**Modern cultivars**	0.57 (0.42–0.79)	0.83 (0.44–1.67)	483 (367–579)	27.0 (18.2–40.5)	5.04 (3.43–6.05)	2.25 (1.97–2.52)	6.55 (6.31–7.03)	9.53 (8.19–10.3)
ANOVA	3.04 **	4.75 **	1.66 *	3.45 **	1.72 *	2.22 **	1.91 *	3.40 **

*, ** significance at 0.05 and 0.01 *p*-levels, respectively.

**Table 2:** Pearson correlation coefficients for secondary metabolites with nutritional and health relevance observed in 156 pea accessions. TPCs = total phenolic compounds; AA = antioxidant activity.

	TPCs	AA	Ssβg	Ss1	Sucrose	Verbascose	Raffinose
**AA**	0.63 **						
**Ssβg**	−0.08	−0.27 **					
**Ss1**	−0.17 *	−0.34 **	0.80 **				
**Sucrose**	0.11	0.13	0.08	0.06			
**Verbascose**	−0.25 **	−0.45 **	0.47 **	0.58 **	0.16		
**Raffinose**	0.05	0.03	0.12	0.23 **	0.55 **	0.39 **	
**Stachyose**	0.07	−0.11	0.27 **	0.39 **	0.41 **	0.64 **	0.72 **

*, ** significance at the 0.05 and 0.01 *p*-levels, respectively.

## Data Availability

The phenotypic and genotypic data used in this work are available at https://doi.org/10.6084/m9.figshare.30898559.

## Supplementary Materials

The following supporting information can be downloaded at: https://www.mdpi.com/article/10.3390/plants15030357/s1. Table S1. Accession name, germplasm pool, area of origin, and material type (modern cultivars vs. landrace/old cultivar) of a worldwide pea germplasm collection, including 156 pea accessions. Table S2. Mean values and Tukey HSD-based mean comparison for traits with nutritional and health relevance observed in 156 pea accessions grouped into 19 landrace/old cultivar germplasm pools and 1 modern cultivar pool. Germplasm pools with different letters differ at *p* < 0.05. Table S3. Genetic coefficient of variation (CVg) for traits with nutritional and health relevance observed in 156 pea accessions grouped into 19 landrace/old cultivar germplasm pools and 1 modern cultivar pool. Table S4. List of genes potentially associated with the significant SNPs detected by GWAS models (BLINK and FarmCPU) based on 10,249 SNPs and performed on 151 accessions for eight traits. Candidate genes were identified by scanning chromosomal regions as far as the distance at which the LD dropped below the threshold for LD decay flanking each significant SNP, and are reported with their annotated function (https://www.pulsedb.org/). TPCs = total phenolic compounds; AA = antioxidant activity. Table S5. Experimental LC-MS/MS data used for tentative identification of saponins in 156 pea (*Pisum sativum*) accessions from different germplasm pools. Figure S1. Cross-entropy criterion for increasing K values based on the SNP data for a germplasm collection of 151 pea accessions. Figure S2. Linkage disequilibrium (LD) decay for each chromosome as squared correlations of allele frequencies (r2) between markers with a maximum distance of 100 Kbp. The X-axis shows the genomic distance in bp. The blue dotted line indicates the intersection between the LOESS curve (red) and half of the average value at the minimal distance (dashed green line), highlighting the value of LD decay in base pairs (bps). Figure S3. Manhattan plots showing the association scores of 10,249 SNPs mapped in the seven pea chromosomes with eight phenotypic traits (TPC, AA, Ssβg, Ss1, sucrose, verbascose, raffinose, and stachyose) characterized in 151 pea accessions (for which SNP data were available). The green continuous line indicates the Bonferroni threshold of significance at 5%. The figure shows the results of a GWAS conducted with the FarmCPU model. Figure S4. Population structure as a PCA performed on the matrix of filtered and imputed SNPs for the 151 pea accessions.

## Abbreviations

The following abbreviations are used in this manuscript:

AA	Antioxidant activity
ACN	Acetonitrile
ANOVA	Analysis of variance
BLUP	Best linear unbiased prediction
rrBLUP	Ridge regression best linear unbiased prediction
DPPH	1,1-diphenyl-2-picrylhydrazyl radical
FA	Formic acid
FT-IR	Fourier transform infrared spectroscopy
FWHM	Full width at half maximum
GAE	Gallic acid equivalent
GBS	Genotyping by sequencing
(U) HPLC	(Ultra)-high-performance liquid chromatography
ISTD	Internal standard
ITT	Ion transfer tube
LD	Linkage disequilibrium
MeOH	Methanol
MS	Mass spectrometry
MS2	Tandem mass spectrometry (fragmentation)
NaOH	Sodium hydroxyde
PVDF	Polyvinylidene fluoride
QC	Quality control
RC	Regenerated cellulose
SNP	Single-nucleotide polymorphism
Ssβg	Soyasaponin βg
Ss1	Soyasaponin I
TE	6-Hydroxy-2,5,7,8-tetramethylchroman-2-carboxylic acid (Trolox) equivalents
TPCs	Total phenolic compounds
