# Supplementary material for: Characterization of Genetic Diversity and Genomic Prediction of Secondary Metabolites in Pea Genetic Resources
Source: Plants (Basel). 2026 Jan 23;15(3):357. doi: 10.3390/plants15030357 (PMC12899914; doi:10.3390/plants15030357)

Supplementary Table S1 Accession name, germplasm pool, area of origin, material type (modern cultivars vs landrace/old cultivar), seed coat and flower colors of a worldwide pea germplasm collection including 156 pea accessions.

Accession name	Germplasm pool	Origin	Type of material	Seed coat color	Flower color
IG49181	Afghanistan	Afghanistan	Landrace/old cultivar	Colored	Purple
IG49189	Afghanistan	Afghanistan	Landrace/old cultivar	Colored	Purple
IG49203	Afghanistan	Afghanistan	Landrace/old cultivar	Colored	Purple
IG52367	Afghanistan	Afghanistan	Landrace/old cultivar	Colored	Purple
IG123021	Afghanistan	Afghanistan	Landrace/old cultivar	Colored	Purple
IG123028	Afghanistan	Afghanistan	Landrace/old cultivar	Colored	Purple
IG123029	Afghanistan	Afghanistan	Landrace/old cultivar	Colored	Purple
IG123030	Afghanistan	Afghanistan	Landrace/old cultivar	NA	NA
IG123041	Central Asia	Kazakhstan	Landrace/old cultivar	Transparent	White
IG123281	Central Asia	Uzbekistan	Landrace/old cultivar	Transparent	White
IG123288	Central Asia	Uzbekistan	Landrace/old cultivar	Colored	White
IG123312	Central Asia	Tajikistan	Landrace/old cultivar	Colored	Purple
IG125597	Central Asia	Tajikistan	Landrace/old cultivar	Colored	Purple
IG125600	Central Asia	Tajikistan	Landrace/old cultivar	Colored	White
IG129009	Central Asia	Kazakhstan	Landrace/old cultivar	NA	NA
IG50669	China	China	Landrace/old cultivar	Colored	Purple
IG52036	China	China	Landrace/old cultivar	Colored	Purple
IG52383	China	China	Landrace/old cultivar	Colored	Purple
IG123227	China	China	Landrace/old cultivar	Colored	Purple
IG123237	China	China	Landrace/old cultivar	Colored	Purple
IG123240	China	China	Landrace/old cultivar	Colored	Purple
IG123245	China	China	Landrace/old cultivar	Colored	Purple
IG134041	East Balkans	Romania	Landrace/old cultivar	NA	NA
IG134060	East Balkans	Romania	Landrace/old cultivar	Colored	Purple
IG134080	East Balkans	Romania	Landrace/old cultivar	Colored	Purple
IG134094	East Balkans	Romania	Landrace/old cultivar	Transparent	White
IG134870	East Balkans	Bulgaria	Landrace/old cultivar	Transparent	White
HRACH Z PARDUBIC	East Balkans	Czechia	Landrace/old cultivar	Transparent	White
LANDRACE ORAVA	East Balkans	Czechia	Landrace/old cultivar	Transparent	White
IG128973	East Balkans	Bulgaria	Landrace/old cultivar	Transparent	White
IG49176	Ethiopia	Ethiopia	Landrace/old cultivar	Colored	Purple
IG51513	Ethiopia	Ethiopia	Landrace/old cultivar	Colored	Purple
IG51516	Ethiopia	Ethiopia	Landrace/old cultivar	Colored	Purple
IG51520	Ethiopia	Ethiopia	Landrace/old cultivar	Colored	Purple
IG51529	Ethiopia	Ethiopia	Landrace/old cultivar	Colored	Purple
IG51540	Ethiopia	Ethiopia	Landrace/old cultivar	NA	NA
IG51562	Ethiopia	Ethiopia	Landrace/old cultivar	Colored	Purple
IG51576	Ethiopia	Ethiopia	Landrace/old cultivar	Colored	Purple
IG52081	Ethiopia	Ethiopia	Landrace/old cultivar	Colored	Purple
IG52092	Ethiopia	Ethiopia	Landrace/old cultivar	Colored	Purple
IG123006	Ethiopia	Ethiopia	Landrace/old cultivar	Colored	Purple
IG123118	Ethiopia	Ethiopia	Landrace/old cultivar	Colored	Purple
IG123136	Ethiopia	Ethiopia	Landrace/old cultivar	Colored	Purple
GLOIRE DE CORREZE	France	France	Landrace/old cultivar	Transparent	White
SERPETTE D'AUVERGNE	France	France	Landrace/old cultivar	Transparent	White
PICAR	France	France	Landrace/old cultivar	Colored	Purple
PIVER	France	France	Landrace/old cultivar	Colored	Purple

SERPETTE DE VITRY	France	France	Landrace/old cultivar	Transparent	White
GRIS DE BOURGOGNE	France	France	Landrace/old cultivar	Colored	Purple
IG49224	Georgia	Georgia	Landrace/old cultivar	Colored	Purple
IG125469	Georgia	Georgia	Landrace/old cultivar	Colored	Purple
IG125472	Georgia	Georgia	Landrace/old cultivar	NA	Purple
IG128856	Georgia	Georgia	Landrace/old cultivar	Colored	Purple
IG134609	Georgia	Georgia	Landrace/old cultivar	Colored	Purple
IG134707	Georgia	Georgia	Landrace/old cultivar	Colored	Purple
IG134828	Georgia	Georgia	Landrace/old cultivar	Colored	Purple
IG134718	Georgia	Georgia	Landrace/old cultivar	Colored	Purple
PIS 845	Germany	Germany	Landrace/old cultivar	Transparent	White
PIS 2242	Germany	Germany	Landrace/old cultivar	NA	NA
IG50559	Greece	Greece	Landrace/old cultivar	Colored	Purple
IG50584	Greece	Greece	Landrace/old cultivar	NA	Purple
IG50641	Greece	Greece	Landrace/old cultivar	Colored	Purple
IG50756	Greece	Greece	Landrace/old cultivar	Colored	Purple
IG50935	Greece	Greece	Landrace/old cultivar	Transparent	White
IG51669	Greece	Greece	Landrace/old cultivar	NA	NA
IG51687	Greece	Greece	Landrace/old cultivar	Colored	Purple
IG51688	Greece	Greece	Landrace/old cultivar	Colored	Purple
IG129002	Greece	Greece	Landrace/old cultivar	Colored	Purple
IG50235	India	India	Landrace/old cultivar	Transparent	White
IG50303	India	India	Landrace/old cultivar	Colored	Purple
IG51891	India	India	Landrace/old cultivar	Transparent	White
IG51927	India	India	Landrace/old cultivar	Transparent	White
IG51948	India	India	Landrace/old cultivar	Transparent	White
IG51957	India	India	Landrace/old cultivar	Transparent	White
IG51993	India	India	Landrace/old cultivar	Colored	Purple
IG51994	India	India	Landrace/old cultivar	Colored	Purple
IG52017	India	India	Landrace/old cultivar	Colored	Purple
IG52050	India	India	Landrace/old cultivar	Colored	Purple
MG 100751	Italy	Italy	Landrace/old cultivar	NA	NA
MG 100948	Italy	Italy	Landrace/old cultivar	Transparent	White
MG 106043	Italy	Italy	Landrace/old cultivar	NA	NA
MG 110243	Italy	Italy	Landrace/old cultivar	Transparent	White
MG 110417	Italy	Italy	Landrace/old cultivar	Transparent	White
MG 110418	Italy	Italy	Landrace/old cultivar	Transparent	White
MG 111850	Italy	Italy	Landrace/old cultivar	Transparent	White
MG 111988	Italy	Italy	Landrace/old cultivar	Transparent	White
MG 115084	Italy	Italy	Landrace/old cultivar	Transparent	White
SPIRALE	Modern cultivar	France	Modern cultivar	Transparent	White
SANTANA	Modern cultivar	Germany	Modern cultivar	Transparent	White
DOVE	Modern cultivar	France	Modern cultivar	Transparent	White
ATTIKA	Modern cultivar	France	Modern cultivar	Transparent	Purple
ISARD	Modern cultivar	France	Modern cultivar	Transparent	White
VIRIATO	Modern cultivar	Spain	Modern cultivar	Transparent	White
CIGARRON	Modern cultivar	Spain	Modern cultivar	Transparent	White
IG114899	Nepal	Nepal	Landrace/old cultivar	NA	White
IG114914	Nepal	Nepal	Landrace/old cultivar	Transparent	White
IG114977	Nepal	Nepal	Landrace/old cultivar	Transparent	Purple
IG115100	Nepal	Nepal	Landrace/old cultivar	Transparent	White

IG115114	Nepal	Nepal	Landrace/old cultivar	Colored	White
IG115228	Nepal	Nepal	Landrace/old cultivar	Transparent	White
IG115266	Nepal	Nepal	Landrace/old cultivar	Transparent	White
IG115331	Nepal	Nepal	Landrace/old cultivar	Colored	Purple
IG115341	Nepal	Nepal	Landrace/old cultivar	Transparent	White
IG112140	North Africa	Morocco	Landrace/old cultivar	Colored	Purple
IG123313	North Africa	Morocco	Landrace/old cultivar	Colored	Purple
IG123004	North Africa	Tunisia	Landrace/old cultivar	Transparent	White
IG125324	Russia	Russia	Landrace/old cultivar	Transparent	White
IG134619	Russia	Russia	Landrace/old cultivar	Transparent	White
IG134621	Russia	Russia	Landrace/old cultivar	Transparent	White
IG134788	Russia	Russia	Landrace/old cultivar	Transparent	White
ZP0076	Spain	Spain	Landrace/old cultivar	Transparent	White
ZP0126	Spain	Spain	Landrace/old cultivar	Transparent	White
ZP0156	Spain	Spain	Landrace/old cultivar	NA	NA
ZP0181	Spain	Spain	Landrace/old cultivar	Transparent	White
ZP0202	Spain	Spain	Landrace/old cultivar	Transparent	White
ZP0798	Spain	Spain	Landrace/old cultivar	Colored	White
ZP1261	Spain	Spain	Landrace/old cultivar	Colored	White
ZP1264	Spain	Spain	Landrace/old cultivar	Colored	Purple
ZP1294	Spain	Spain	Landrace/old cultivar	Colored	White
ZP1300	Spain	Spain	Landrace/old cultivar	Transparent	White
IG50248	Turkey	Turkey	Landrace/old cultivar	Colored	Purple
IG50250	Turkey	Turkey	Landrace/old cultivar	Transparent	White
IG50358	Turkey	Turkey	Landrace/old cultivar	Colored	Purple
IG50362	Turkey	Turkey	Landrace/old cultivar	Colored	Purple
IG52521	Turkey	Tyrkey	Landrace/old cultivar	Colored	Purple
IG116297	Turkey	Turkey	Landrace/old cultivar	Colored	Purple
IG116369	Turkey	Turkey	Landrace/old cultivar	NA	NA
IG123050	Turkey	Turkey	Landrace/old cultivar	Transparent	White
ENGLISH WONDER	UK	Great Britain	Landrace/old cultivar	Transparent	White
EMERALD GEM	UK	Great Britain	Landrace/old cultivar	Transparent	White
TELEGRAPH	UK	Great Britain	Landrace/old cultivar	NA	NA
KENTISH INVICTA	UK	Great Britain	Landrace/old cultivar	Transparent	White
ALDERMAN	UK	Great Britain	Landrace/old cultivar	Transparent	White
MAGNUM BONUM	UK	Great Britain	Landrace/old cultivar	Transparent	White
KNIGHTS MARROW	UK	Great Britain	Landrace/old cultivar	Transparent	White
KNIGHTS DWARF WHITE	UK	Great Britain	Landrace/old cultivar	Transparent	White
ENGLISH MAPLE	UK	Great Britain	Landrace/old cultivar	Colored	Purple
IG124843	Ukraine	Ukraine	Landrace/old cultivar	Transparent	White
IG125336	Ukraine	Ukraine	Landrace/old cultivar	Transparent	White
IG128863	Ukraine	Ukraine	Landrace/old cultivar	Colored	Purple
IG128913	Ukraine	Ukraine	Landrace/old cultivar	Transparent	White
IG128934	Ukraine	Ukraine	Landrace/old cultivar	Transparent	White
IG134744	Ukraine	Ukraine	Landrace/old cultivar	Transparent	White
IG134746	Ukraine	Ukraine	Landrace/old cultivar	Transparent	White
IG134750	Ukraine	Ukraine	Landrace/old cultivar	Transparent	White
IG134770	Ukraine	Ukraine	Landrace/old cultivar	Transparent	White
IG134772	Ukraine	Ukraine	Landrace/old cultivar	Transparent	White
IG52534	West Asia	Syria	Landrace/old cultivar	Colored	Purple

IG52535	West Asia	Syria	Landrace/old cultivar	Colored	Purple
IG123080	West Asia	Palestine	Landrace/old cultivar	Colored	Purple
IG52401	West Asia	Syria	Landrace/old cultivar	Colored	Purple

NA = not available.

Supplementary Table S2. Mean values and Tukey HSD-based mean comparison for traitswith nutritional and health relevance observed in 156 pea accessions grouped into 19 landrace/old cultivar germplasm pools and one modern cultivar pool. Germplasm pools with different letter differ at $P < 0.05$.

Germplasm.pool	TPC (mg GAE/g)	AA (µmol TE/g)	Ssβg	Ss1	Sucrose	Raffinose	Stachyose	Verbascose
**Afghanistan**	0.80 (d)	1.35 (bcd)	500 (ab)	21.4 (a)	6.36 (ab)	2.16 (ab)	6.44 (ab)	6.73 (a)
**Central Asia**	0.65 (abcd)	0.83 (abc)	466 (ab)	21.6 (a)	5.87 (ab)	2.35 (ab)	6.79 (ab)	8.54 (ab)
**Cina**	0.65 (abcd)	1.17 (abcd)	571 (ab)	27.1 (a)	8.08 (b)	2.66 (ab)	6.84 (ab)	7.70 (a)
**East Balkans**	0.54 (a)	0.62 (a)	545 (ab)	32.3 (ab)	5.80 (ab)	2.47 (ab)	6.89 (ab)	9.13 (ab)
**Ethiopia**	0.74 (bcd)	1.37 (cd)	584 (ab)	26.9 (a)	5.32 (ab)	2.17 (ab)	6.08 (a)	8.29 (a)
**France**	0.69 (abcd)	1.27 (abcd)	331 (a)	18.7 (a)	6.35 (ab)	2.66 (ab)	6.31 (ab)	7.45 (a)
**Georgia**	0.70 (abcd)	1.18 (abcd)	381 (ab)	19.1 (a)	5.97 (ab)	2.17 (ab)	6.34 (ab)	7.04 (a)
**Germany**	0.62 (abcd)	0.60 (abcd)	696 (ab)	36.1 (ab)	5.70 (ab)	2.85 (ab)	6.62 (ab)	9.15 (ab)
**Greece**	0.75 (bcd)	1.37 (bcd)	501 (ab)	23.4 (a)	6.29 (ab)	2.55 (ab)	6.79 (ab)	7.48 (a)
**India**	0.68 (abcd)	1.03 (abcd)	503 (ab)	26.3 (a)	5.59 (ab)	2.29 (ab)	6.72 (ab)	8.42 (a)
**Italy**	0.67 (abcd)	0.93 (abc)	373 (ab)	22.6 (a)	5.19 (ab)	2.91 (ab)	7.56 (ab)	9.70 (ab)
**Nepal**	0.59 (abc)	0.79 (abc)	512 (ab)	27.6 (a)	4.98 (ab)	2.37 (ab)	6.88 (ab)	9.26 (ab)
**North Africa**	0.72 (abcd)	1.36 (abcd)	440 (ab)	29.9 (ab)	5.06 (ab)	2.06 (b)	6.52 (ab)	7.65 (ab)
**Russia**	0.62 (abcd)	0.71 (abc)	484 (ab)	29.8 (ab)	5.51 (ab)	2.33 (ab)	6.18 (ab)	8.00 (ab)
**Spain**	0.66 (abcd)	0.94 (abc)	531 (ab)	28.0 (a)	6.04 (ab)	2.79 (ab)	7.07 (ab)	10.00 (ab)
**Turkey**	0.67 (abcd)	1.30 (bcd)	436 (ab)	21.0 (a)	7.20 (ab)	2.33 (ab)	6.14 (ab)	7.06 (a)
**UK**	0.58 (ab)	0.73 (ab)	659 (b)	47.4 (b)	5.97 (ab)	2.75 (ab)	7.99 (b)	11.74 (b)
**Ukraine**	0.68 (abcd)	0.63 (a)	532 (ab)	26.9 (a)	4.33 (a)	2.07 (ab)	6.19 (ab)	8.02 (a)
**West Asia**	0.83 (cd)	1.82 (d)	517 (ab)	24.5 (a)	7.32 (ab)	3.16 (a)	7.92 (ab)	9.40 (ab)
**Modern cultivars**	0.57 (abc)	0.83 (abc)	483 (ab)	26.9 (a)	5.04 (ab)	2.25 (ab)	6.55 (ab)	9.53 (ab)

TPC = Total phenolic compounds; AA = Antioxidant activity; GAE = Gallic acid equivalents; TE = 6-Hydroxy-2,5,7,8-tetramethylchroman-2-carboxylic acid (Trolox) equivalents.

Supplementary Table S3. Genetic coefficient of variation (*CVg*) for traitswith nutritional and health relevance observed in 156 pea accessions grouped into 19 landrace/old cultivar germplasm pools and one modern cultivar pool.

Germplasm.pool	TPC	AA	Ssβg	Ss1	Sucrose	Raffinose	Stachyose	Verbascose	Pool mean CVg
**Afghanistan**	19.22	44.38	36.42	38.97	0	19.2	12.35	30.83	25.17
**Central Asia**	10.21	30.55	35.8	22.48	21.79	24.97	16.42	17.19	22.43
**Cina**	8.02	18.23	11.09	19.36	23.6	16.72	13.68	20.35	16.38
**East Balkans**	23.68	50.15	28.59	40.62	0	28.41	20.08	19.3	26.35
**Ethiopia**	13.49	24.41	29.2	24.31	12.58	13.68	10.3	14.58	17.82
**France**	17.81	25.8	40.44	36.18	39.27	33.8	13.59	25.2	29.01
**Georgia**	15.92	36.06	40.79	37.03	16.84	22.84	13.47	27.4	26.29
**Germany**	5.37	25.41	0	6.13	0	5.88	2.15	7.7	6.58
**Greece**	22.41	31.29	40.66	40.37	22.64	21.38	10.39	30.4	27.44
**India**	20.1	47.62	25.2	38.64	0	18	7.01	13.22	21.22
**Italy**	11.09	17.05	51.2	37.97	31.26	23.36	22.99	24.79	27.46
**Nepal**	23.37	52.92	35.03	25.69	0	18.11	12.2	4.94	21.53
**Nord Africa**	16.25	32.37	7.36	38.42	0	11.99	12.88	0	14.91
**Russia**	14.26	15.78	16.19	18.63	0	6.45	10.82	21.48	12.95
**Spain**	12.83	50.19	37.32	42.66	38.13	23.94	22.78	17.95	30.72
**Turkey**	15.43	37.48	59.69	53.3	13.84	21.53	14.73	42.93	32.37
**UK**	13.22	60.06	36.46	37.37	0	22.11	26.54	29.59	28.17
**Ukraine**	19.55	50.37	24.64	28.6	0	14.74	10.29	16.39	20.57
**West Asia**	13.12	9.76	29.25	28.69	15.91	0	7.6	9.03	14.17
**Modern cultivars**	18.7	47.78	16.31	26.03	0	8.15	1.98	6.08	15.63
**Trait mean CVg**	15.7	34.73	30.1	32.07	11.79	17.76	13.11	18.97	

TPC = Total phenolic compounds; AA = Antioxidant activity.

Supplementary Table S4. List of genes potentially associated to the significant SNPs detected by GWAS models (BLINK and FarmCPU.) based on 10,249 SNPs and performed on 151 accessions for eight traits. Candidate genes were identified scanning chromosomal regions as far as the distance at which LD dropped below the threshold for LD decay flanking each significant SNP and are reported with their annotated function (https://www.pulsedb.org/). TPC = Total phenolic compounds; AA = Antioxidant activity.

SNP	Trait	Model	% Var. Expl.	Gene	Function	Comments
chr1LG6_62867436	Ssβg	FarmCPU.	0.02	Psat1g040440.1	Sulfate permease family	OBS: Chr1 LD
chr3LG5_92532995	Ssβg	FarmCPU.	0.01	Psat3g042320.1	ABC transporter transmembrane region (mRNA)	SNP inside gene
chr5LG3_221343164	Ssβg	FarmCPU.	2.55	Psat5g124120.1	Enoyl-(Acyl carrier protein) reductase	SNP inside gene
chr6LG2_23631855	Ssβg	FarmCPU.	0.01	No gene in LD		NO
chr7LG7_22491409*	Ssβg	FarmCPU.	2.85	Psat7g014680.1	Cytidine and deoxycytidylate deaminase zinc-binding region	SNP inside gene
chr7LG7_22491409*	Ssβg	BLINK.		..	..	..
chr1LG6_137521369	Ss1	FarmCPU.	0.01	Psat1g084240	UBX domain	SNP inside gene
chr2LG1_83094492	Ss1	FarmCPU.	2.51	Psat2g047960.2	Binding (mRNA)	SNP inside gene
chr2LG1_415406607*	Ss1	FarmCPU.	0.02	Psat2g176120.1	Vesicle-associated membrane protein	SNP inside gene
chr2LG1_415406607*	Ss1	BLINK.		..	..	..
chr5LG3_248573284*	Ss1	FarmCPU.	0.03	Psat5g137080.1	Acetyl-CoA carboxylase + central region	SNP inside gene
chr5LG3_248573284*	Ss1	BLINK.		..	..	..
chr5LG3_105360859	TPC	BLINK.	8.14	Psat5g058000.1	Protein of unknown function (DUF668)	SNP inside gene
chr6LG2_41547969	TPC	BLINK.	2.23	Psat6g046520.1	Ribosomal protein S5 + N-terminal domain	SNP inside gene
chr7LG7_121841485	TPC	BLINK.	0.19	Psat7g072560.1	Peptidyl-tRNA hydrolase	SNP inside gene
chr7LG7_362552098	TPC	BLINK.	1.16	Psat7g192120.1	Protein kinase domain	SNP inside gene
chr7LG7_489760431	TPC	BLINK.	5.52	Psat7g263160.1	Plastocyanin-like domain	SNP inside gene
chr3LG5_26270285	AA	FarmCPU.	5.38	Psat3g011000.1	PPR repeat family (mRNA)	SNP inside gene
chr4LG4_281868801	AA	FarmCPU.	0.01	Psat4g143520.1	Pectinesterase	SNP inside gene
chr4LG4_436873231	AA	FarmCPU.	0.05	Psat4g217880.1	Subtilase family (mRNA)	SNP inside gene
chr5LG3_107738991*	AA	FarmCPU.	0.02	Psat5g059320.1	Transmembrane amino acid transporter protein	SNP inside gene
chr5LG3_107738991*	AA	BLINK.		..	..	..
chr6LG2_68261112	AA	BLINK.	6.17	Psat6g060320.1	EF hand associated	SNP inside gene
chr6LG2_417288431	AA	BLINK.	8.96	Psat6g209120.1	Thi4 family	SNP inside gene
chr7LG7_138894295	AA	FarmCPU.	0.03	Psat7g082760.1	Zein-binding	SNP inside gene
chr1LG6_17453550	Verbascose	FarmCPU.	0.01	Psat1g013760.1	Rhodanese-like domain	SNP inside gene
chr3LG5_95340437	Verbascose	FarmCPU.	0.02	Psat3g042640.1	Histidyl-tRNA synthetase (mRNA)	SNP inside gene
chr3LG5_44073760**	Verbascose	BLINK.	4.32	Psat3g019760.1	FACT complex subunit (SPT16/CDC68) (mRNA)	SNP inside gene
chr5LG3_404955801	Verbascose	FarmCPU.	0.09	Psat5g199360.1	Response regulator receiver domain	SNP inside gene
chr5LG3_542182751	Verbascose	FarmCPU.	0.68	Psat5g278320.1	Thioredoxin	SNP inside gene

chr7LG7_89997988	Verbascose	FarmCPU.	1.84	Psat7g053680.1	SCO1/SenC	SNP inside gene
chr7LG7_428666716	Verbascose	FarmCPU.	0.02	Psat7g213760.1	ATPase family associated with various cellular activities (AAA)	SNP inside gene
chr1LG6_17378315	Stachyose	FarmCPU	0.01	Psat1g013480.1	Peroxidase	SNP inside gene
chr1LG6_77496792	Stachyose	BLINK.	1.53	Psat1g049720.1	Mitochondrial carrier protein signature	SNP inside gene
chr3LG5_285621982	Stachyose	FarmCPU.	0.02	Psat3g148200.1	CDC45-like protein (mRNA)	SNP inside gene
chr3LG5_44073760**	Stachyose	FarmCPU.	4.32	..	..	..
chr3LG5_44073760**	Stachyose	BLINK.		..	..	..
chr7LG7_13331802	Stachyose	FarmCPU.	0.05	Psat7g008240.1	Unknown gene	
chr7LG7_77652530	Stachyose	FarmCPU.	0.01	Psat7g045040.1	AIR synthase related protein + C-terminal domain	SNP inside gene
chr4LG4_52663740	Raffinose	BLINK.	1.23	Psat4g035920.1	Unknown gene (mRNA)	SNP inside gene
chr5LG3_296150921	Raffinose	BLINK	2.68	Psat5g162200.1	Radical SAM ThiC family	SNP inside gene
chr4LG4_439662725	Sucrose	BLINK	4.34	Psat4g219720.1	Unknown gene	SNP inside gene

*SNPs identified by both BLINK and FarmCPU models, **SNPs associated to more than a trait.

Supplementary Table S5. Experimental LC-MS/MS data used for tentative identification of saponines in 156 pea (Pisum satitum) accessions from different germplasm pools.

Compound	MF	RT (min)	Parent *m/z* (M-H)[-]	Δ *m/z* (ppm)	Product ions (intensity %) at CID 30 %
Ginsenoside (ISTD)	$C_{54}H_{92}O_{23}$	8.25	1107.5956	0.1	945.5433 (100), 783.4911 (36), 621.4362 (10)
Soyasaponin βg	$C_{54}H_{84}O_{21}$	10.85	1067.5432	0	1049.5319 (100), 741.4233 (49), 1035.5159 (49), 967.5255 (44), 879.5105 (37)
Soyasaponin I	$C_{48}H_{78}O_{18}$	9.85	941.5115	0	923.4998 (100), 879.5099 (63), 733.4521 (52), 615.3890 (41), 525.3936 (18), 597.3784 (18), 457.3676 (12), 795.4520 (11)

Supplementary Figure S1. Cross-entropy criterion for increasing K values based on the SNPs data for a germplasm collection of 151 pea accessions.

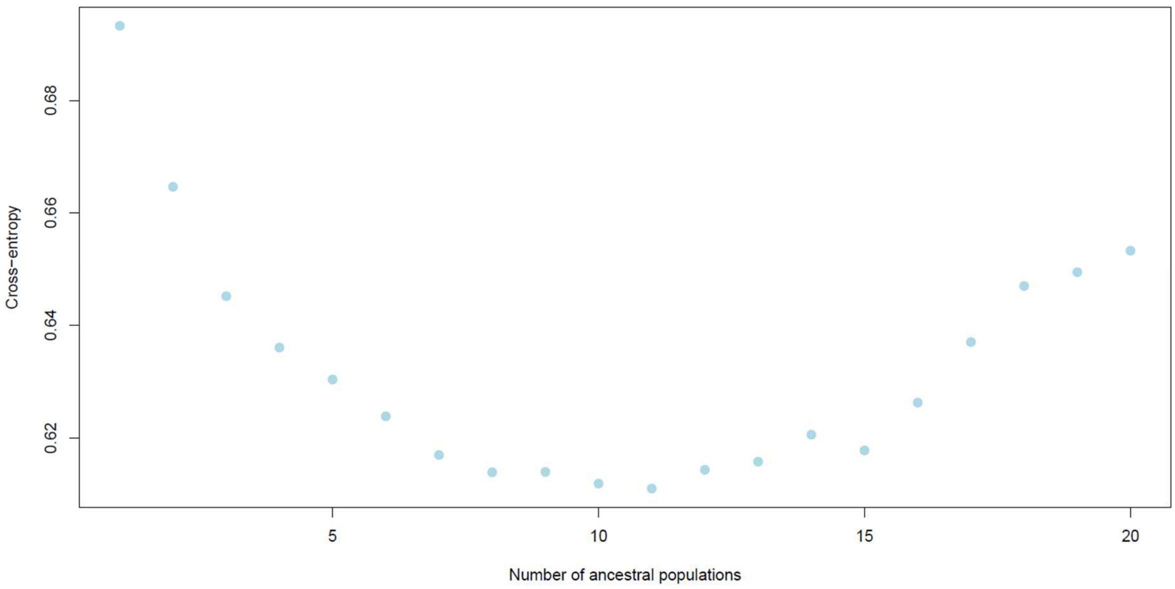

Supplementary Figure S2. Linkage disequilibrium (LD) decay for each chromosome as squared correlations of allele frequencies (r2) between markers with a maximum distance of 100 Kbp. The X-axis shows the genomic distance in bp. The blue dotted line indicates the intersection between the LOESS curve (red) and the half of the average value at the minimal distance (dashed green line), highlighting the value of LD decay in base pairs (bp).

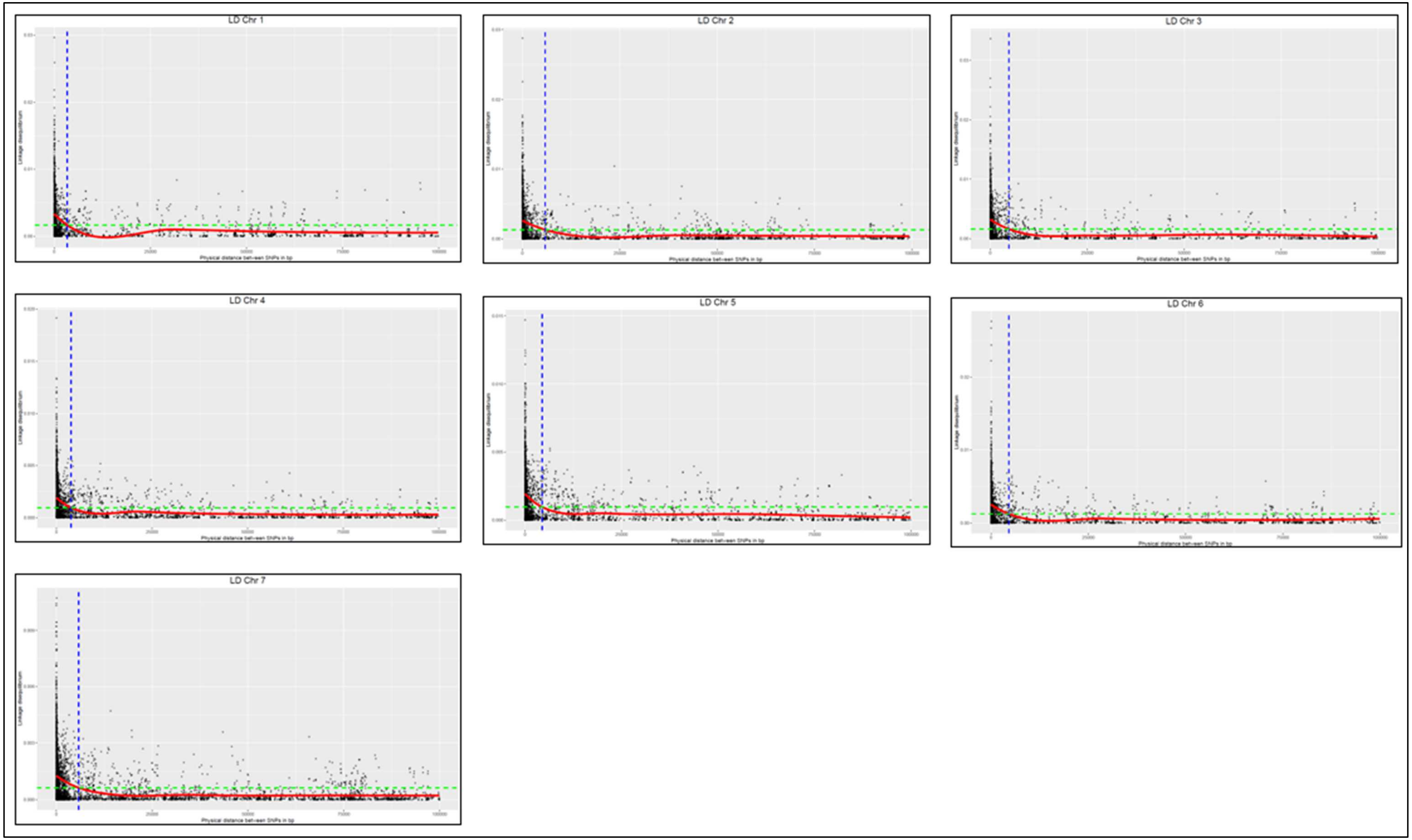

Supplementary Figure S3. Manhattan plots showing the association scores of 10,249 SNPs mapped in the seven pea chromosomes with eight phenotypic traits (TPC, AA, Ssβg, Ss1, sucrose, verbascose, raffinose and stachyose) characterized in 151 pea accessions (for which SNPs data were available). The green continuous line indicates the Bonferroni threshold of significance at 5%. The figure shows the results of GWAS conducted with the FarmCPU model.

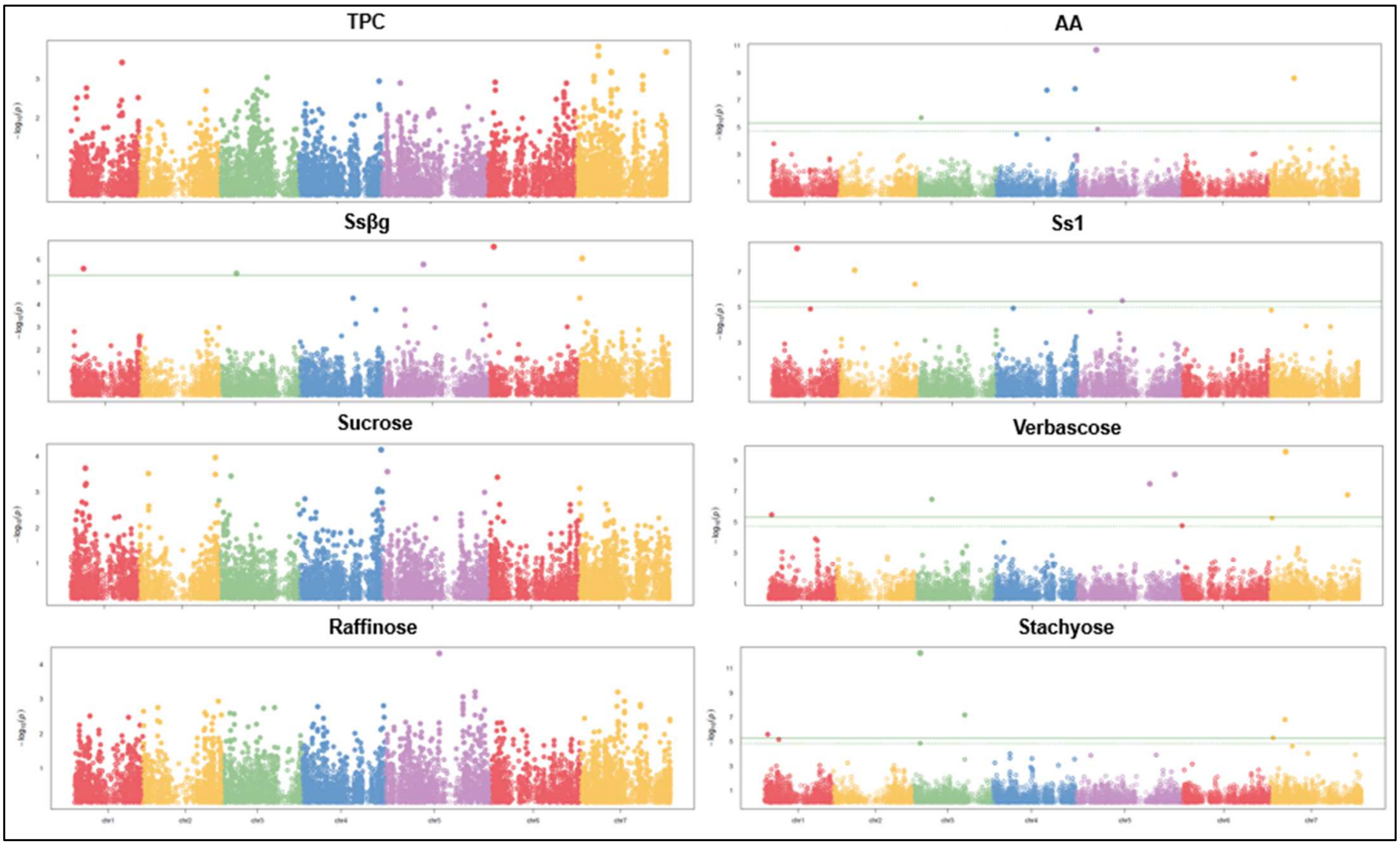

Supplementary Figure S4. Population structure as PCA performed on the matrix of filtered and imputed SNPs for the 151 pea accessions.

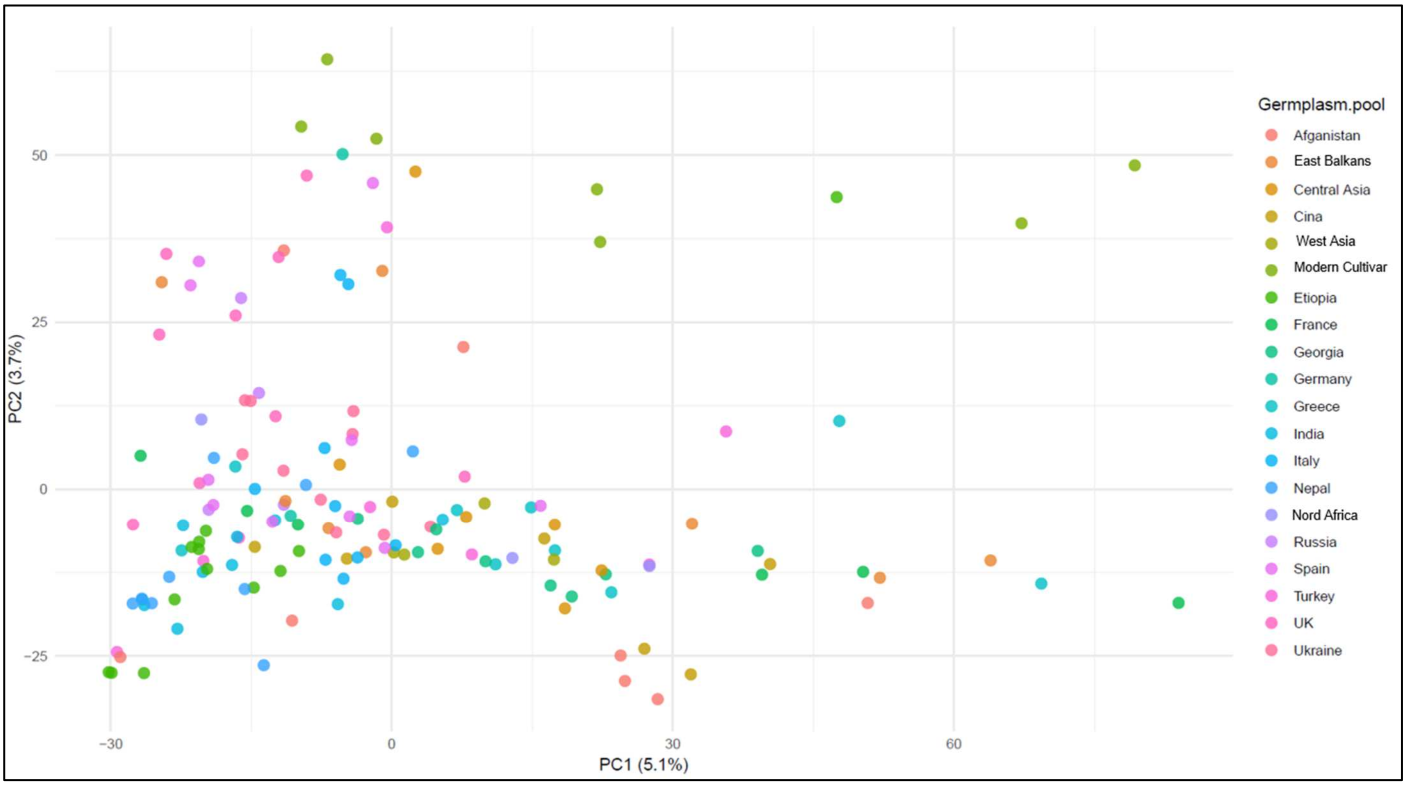

Supplement: Supplementary file 1 [file plants-15-00357-s001.zip › plants-4085803-supplementary.pdf]
